# Fever enhances host bacterial defence while limiting mitochondrial damage

**DOI:** 10.1038/s41467-026-78498-6

**Published:** 2026-10-14

**Authors:** Yonghan Wu, Elijah Rowe, Albert Siryaporn, Steven P. Gross

**Affiliations:** 1 Department of Physics and Astronomy, University of California Irvine, Irvine, CA USA; 2 Department of Molecular Biology and Biochemistry, University of California Irvine, Irvine, CA USA; 3 Department of Developmental and Cell Biology, University of California Irvine, Irvine, CA USA

**Keywords:** Antimicrobial responses, Innate immunity, Microbiology

## Abstract

Infection triggers the fever response, but the response’s effect on antimicrobial peptide (AMP) function remains unknown. We discovered that human AMP LL-37 antibacterial activity is temperature-dependent and were curious as to why. Its temperature dependence is not an accident, as AMPs from other animals with different body temperatures have distinct temperature sensitivities. We find that AMP temperature sensitivities are tuned to animals’ body temperatures, and that for animals able to raise their body temperature, this is used to increase the AMP’s antibacterial efficacy. This effect reflects a balance between optimizing antibacterial killing while minimizing mitochondrial damage. In contrast, cold-blooded animals that are unable to raise their body temperature use a different strategy to avoid mitochondrial damage. Together, this study suggests that fever is beneficial for antimicrobial defense by raising AMP activity towards bacteria while reducing mitochondrial damage when pathogens are absent.

## Abstract (web summary)

Animals’ antimicrobial peptides (AMPs) are evolutionarily tuned for their body temperatures, but vertebrates’ fever boosts their AMPs' killing ability. We show this reflects a trade-off to avoid mitochondrial damage.

## Introduction

Animals have evolved distinct thermoregulatory strategies. Mammals maintain their body temperatures around 37 °C, whereas amphibians and reptiles allow the environment to adjust their body temperature to a temperature typically lower than that of mammals. Following infection, the body temperature of mammals rises as part of the fever response. Snakes, while cold-blooded, fall in between, as they can actively raise their body temperature by basking under sunlight^1^, while most other cold-blooded animals lack this ability. Both the innate and adaptive immune systems are stimulated by elevated temperature^2^. However, how elevated temperature leads to improved clearance of pathogens in hosts is not understood at the molecular level^3^. Bacterial pathogens contain heat shock systems that make them tolerant to elevated temperatures^4^, and some pathogens grow faster at elevated temperatures^5,6^. Furthermore, higher temperatures have significant detrimental effects on host cells, including impairing mitochondrial activity through uncoupling oxidative phosphorylation^7,8^. Given the many potential downsides of elevated temperatures, it is unclear how the strategy could improve host survival.

One important component of innate immunity is antimicrobial peptides (AMPs), which provide a key defense against bacterial infection^9–12^. The effect of temperature on AMPs’ activity is not understood. In the case of bacteriocin AMPs, an increase in temperature decreases the antimicrobial effects of the antimicrobial peptide^13^. For the chickpea AMP Leg1, antimicrobial activity is insensitive to temperature over a broad range of 4 °C to 37 °C^14^. Contributing to the knowledge gap is that most studies on AMPs’ activity are performed at human physiological temperature at 37 °C^15,16^ rather than at the host’s baseline body temperature. Since elevated temperatures can destabilize peptide structure, induce structural transitions, or non-specifically alter protein activity^17–20^, elevated temperatures during a fever response would not be expected to produce greater microbial killing.

The human AMP cathelicidin (LL-37) permeabilizes bacterial membranes^21,22^ and is found in neutrophil extracellular traps (NETs) and on lipid droplets, where it colocalizes with histones^23–25^. Histones also exhibit antimicrobial activity^26^ and show synergistic activity with AMPs^10,27–29^. Previous synergy studies have shown that a combined treatment of histones and LL-37 induces durable bactericidal pore formation while AMPs alone do not^28^. To better understand the synergy between LL-37 and histones, we carefully investigated factors that affect AMP activity under their physiologically relevant concentrations.

We explored the utility of such temperature dependence and suggest that it is not an accident, but rather reflects a careful balance between optimizing for antibacterial killing while avoiding mitochondrial damage. We observed that the antimicrobial activity of LL-37 is relatively low slightly below room temperature (22 °C), elevated at human body temperature (37 °C), and highest at febrile temperature (40 °C). We then tested AMPs from cold-blooded animals such as fish and frogs and found that they all show optimal activity at lower temperatures close to their animals’ living environment. This result suggests that the temperature-dependent activity is due to an evolutionary tuning of the innate immune system rather than faster reaction kinetics. Our data also suggest that it is possible to evolutionarily tune the AMPs’ relative efficacy against bacteria versus mitochondria. At febrile temperature, in human lung and liver cells, we observed decreased contact between lipid droplets and mitochondria, which may protect mitochondria from damage caused by AMPs and histones on lipid droplets. For animals lacking the ability to effect changes in body temperature, a different evolutionary strategy appears to be employed, where AMPs undergo different tuning: rather than increasing their temperature-dependent damage, they likely evolved to decrease mitochondrial damage. In this case, the cellular mitochondria-LD interaction appears temperature-insensitive.

## Results

### Antimicrobial synergy is amplified by increasing temperature

**Fig. 1:**
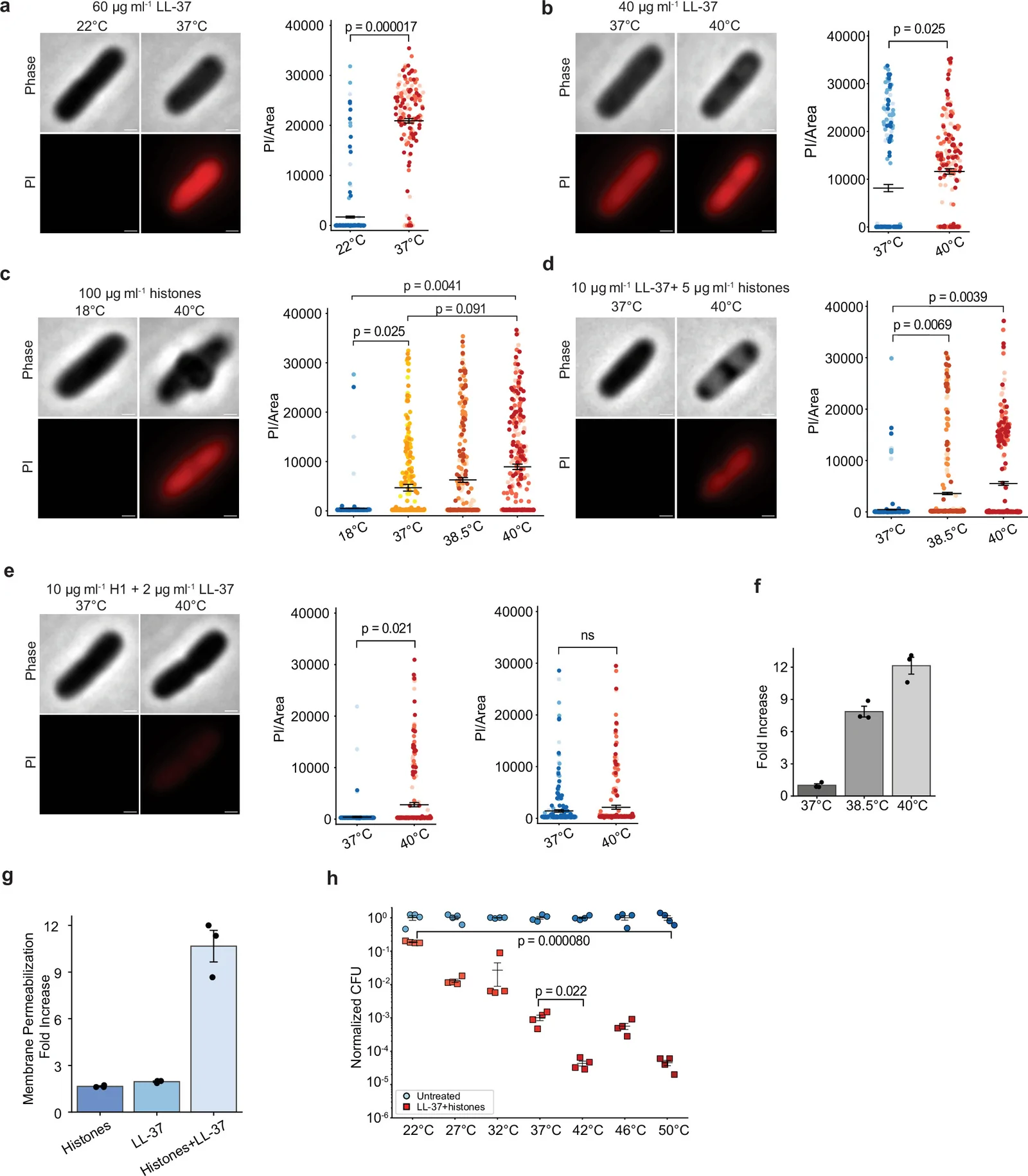
Febrile temperatures increase the antimicrobial activity of human LL-37, histones, and dual treatments. **a** Representative phase-contrast and fluorescence images of *E. coli* MG1655 treated with 60 μg ml^−1^ LL-37 slightly below room temperature (22 °C) and at physiological temperature (37 °C). Propidium iodide (PI) fluorescence is quantified and plotted for each cell. **b** 40 μg ml^−1^ LL-37 treatment at 37 °C and 40 °C. **c** 100 μg ml^−^^1^ histones (containing a mixture of core and linker histones) at a range of temperatures including below room temperature, physiological temperature, and febrile temperatures. **d** 10 μg ml^−^^1^ LL-37 and 5 μg ml^−^^1^ histones at physiological temperature and febrile temperatures. **e** 2 μg ml^−^^1^ LL-37 and 10 μg ml^−^^1^ histone H1 (left) and 400 μg ml^−^^1^ histone H1 alone (right) at physiological and febrile temperatures. PI was added after treatment to assess membrane permeability. **f** Fold increases of PI fluorescence at different temperatures following 1 h dual treatment using 5 μg ml^−1^ histones and 10 μg ml^−1^ LL-37, normalized by the fluorescence at 37 °C. **g** Fold increase in the fraction of cells with PI entry at 40 °C compared to 37 °C, for treatment with 100 μg ml^−^^1^ histones, 40 μg ml^−^^1^ LL-37 and 10 μg ml^−^^1^ LL-37 and 5 μg ml^−^^1^ histones. **h** CFU assays performed at indicated various temperatures for treatment using 10 μg ml^−1^ histones and 40 μg ml^−1^ LL-37, normalized to the untreated condition for each temperature. Four biological replicates were performed for each condition. All treatments were performed for 1 h. For the PI experiment, three biological replicates were performed for each condition, and each replicate was plotted in a different color. 100 cells were imaged in each replicate. Each data point represents an individual cell. Black bars indicate the mean, and error bars represent the standard error of the mean (SEM) of three biological replicates. Scale bars represent 0.5 μm. Two-sided Welch’s t-tests with unequal variances were performed. No adjustments were made for multiple comparisons. *P*-values greater than 0.05 are denoted as non-significant (ns).

We tested histones and LL-37 alone and in conjunction with each other, at a range of temperatures (Fig. 1a–d). Core and linker histones were both present in the histones sample. Circulating histone concentrations reach 3 µg ml^−1^ in sepsis patients^30^ and can exceed 50 µg ml^−1^ in acute respiratory distress syndrome (ARDS) patients^31^, whereas LL-37 concentrations range from 5 µg ml^−1^ to 30 µg ml^−1^ in healthy infants or during infections, respectively^32,33^. At these concentrations, LL-37 and histones give rise to antimicrobial synergy through membrane pore formation^28^. The molecules were used individually at higher concentrations in order to achieve comparable membrane permeabilization. The activity of the molecules was measured using two methods: by their ability to permeabilize *E. coli* membranes measured through propidium iodide (PI) entry, and by their ability to kill bacteria, assessed by colony-forming unit (CFU) assays. We found that LL-37’s activity was relatively low at sub-physiological body temperatures, increased at body temperature (37 °C), and was significantly higher at a febrile temperature of 40 °C (Fig. 1a, b). While these assays were performed in bacterial growth medium, the temperature effect was also observed in DMEM media, which more closely replicates a host environment (Supplementary Fig. 1a). The trend of greater PI entry with greater temperature was also observed for histones alone (Fig. 1c) and for the LL-37/histones and LL-37/histone H1 dual treatments (Fig. 1d, e). H1 is a linker histone and has previously been demonstrated to have antimicrobial activity^34,35^ and was the only histone present in the histone H1 sample. Notably, there was a massive 12-fold increase in the mean PI fluorescence of the synergistic LL-37/histone dual treatment at 40 °C vs. 37 °C, and at 38.5 °C the increase was only a slightly less increase at 8-fold (Fig. 1d, f). The increase in membrane permeabilization at febrile temperature is significantly greater for dual treatment than for histones or LL-37 alone (Fig. 1g), indicating an advantage of synergy. Temperature increases from room temperature to normal body and febrile temperatures resulted in significant increases in bactericidal activity as assayed by CFUs (Fig. 1h). Consistent with this trend, prolonged treatment for 4 h instead of 1 h showed complete killing at 38.5 °C while only partial killing at 37 °C (Supplementary Fig. 1b). Thus, the fever response dramatically increases bactericidal activity.

**Fig. 2:**
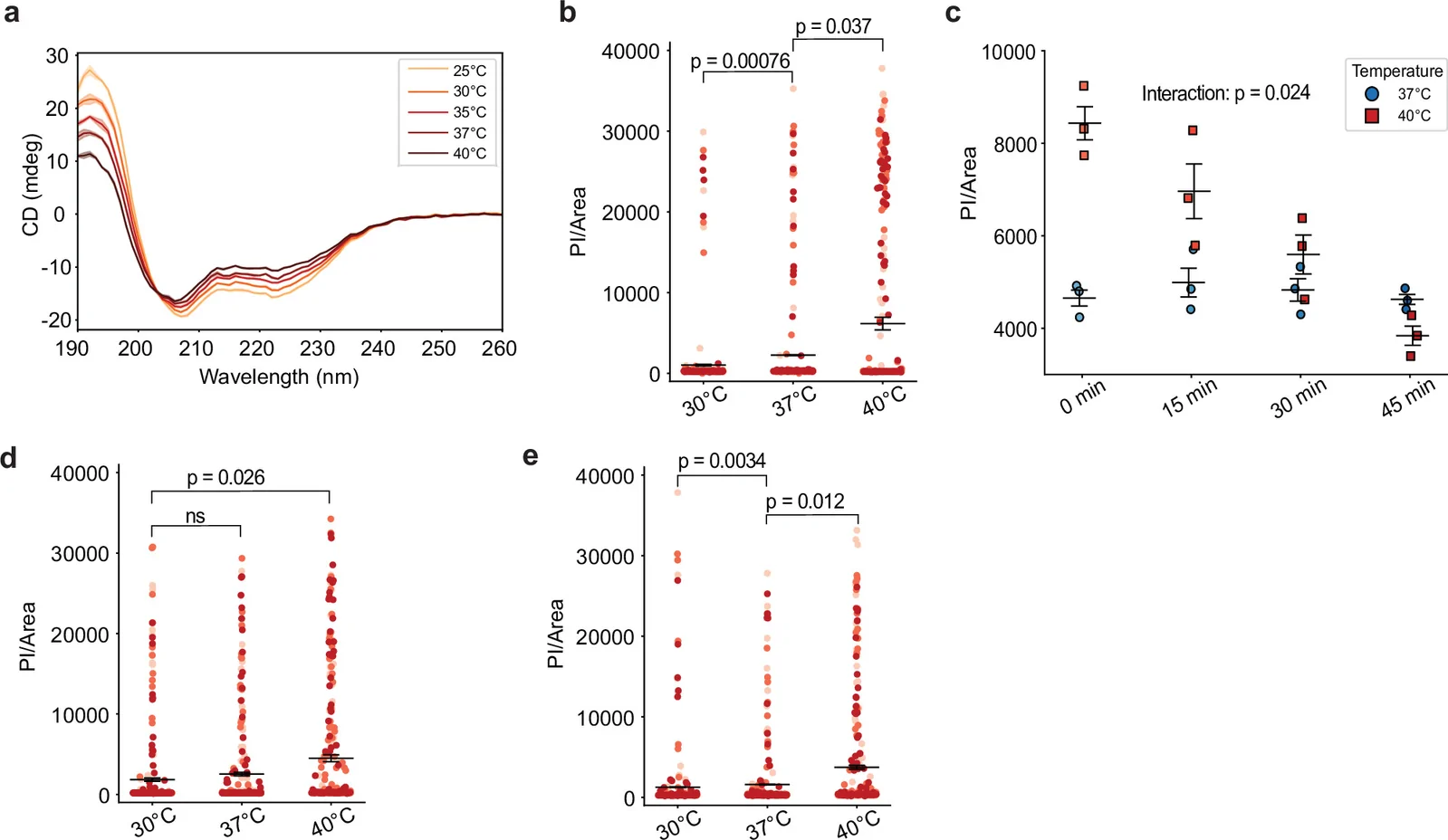
Structural characterization and hysteretic antimicrobial activity of human LL-37. **a** Far UV circular dichroism (CD) spectra of 0.125 mg ml^−1^ LL-37 in MinA medium at temperatures of 25 °C and 40 °C. Four technical replicates were performed at 25 °C; five technical replicates were performed at 30 °C, 35 °C, and 37 °C; six replicates were performed at 40 °C. Solid lines represent the mean, and shaded regions show the SEM. **b** PI fluorescence from three biological replicates of *E. coli* treated at 37 °C with 20 μg ml^−1^ LL-37 that were preincubated without *E. coli* at the indicated temperatures for 1 h before treating *E. coli* that were cultured separately at 37 °C and subsequently incubated at 37 °C for 10 min. Data points represent individual *E. coli* and each replicate is plotted as a different color. **c** PI fluorescence from three biological replicates of *E. coli* treated with 20 μg ml^−1^ LL-37 that was preincubated without *E. coli* at 37 °C or 40 °C for 1 h, incubated for the indicated additional times at 37 °C, and then added to *E. coli* at 37 °C for 10 min. Data points represent the average PI/area from each replicate. Two-way ANOVA excluding 0 min measurements revealed a significant temperature and time interaction (*P* = 0.0236). PI quantification of E. coli MG1655 at 37 °C that were treated with 100 μg ml^−1^ histones (**d**) or 5 μg ml^−1^ histones plus 5 μg ml^−1^ LL-37 (**e**) that were preincubated without bacteria for 1 h at the indicated temperatures and then added to bacteria at 37 °C for 10 min. For the PI experiment, three biological replicates were performed for each condition and plotted as different color densities. At least 100 cells were imaged in each replicate. Black bars indicate the mean, and error bars represent SEM from three replicates. Two-sided Welch’s t-tests with unequal variances were performed. No adjustments were made for multiple comparisons. *P*-values greater than 0.05 are denoted as non-significant (ns).

Mechanistically, how might this functional change occur? We found that increases in temperature alter the LL-37 secondary structure content (Fig. 2a). Although LL-37 showed α-helical bands at all temperatures tested, the amplitude of each band, which is often used to estimate helical content^36–38^, shifted with temperature. Specifically, the α-helical content decreased from 45.8% at 25 °C to 34.0% at 37 °C and to 29.5% at 40 °C. Furthermore, the estimated antiparallel β-sheet content increased from 3.1% to 9.0% and to 11.4% at 25 °C, 37 °C, and 40 °C, respectively. This suggests that the greater efficacy arises from changes in LL-37 itself. A previous study showed that the bacterium *Yersinia enterocolitica* produces a temperature-dependent response to antimicrobial peptides^39^. However, in our experiment the greater efficacy is not due to changes in the bacterial physiology at the different temperatures, as pre-incubation of LL-37 at different temperatures was sufficient to alter its activity towards bacteria that were cultured and treated at a fixed temperature of 37 °C (Fig. 2b). Notably, LL-37 retained its altered antimicrobial activity after being transferred from 40 °C to 37 °C, returning to its original activity at 37 °C within 45 minutes (Fig. 2c), indicating that LL-37 exhibits distinct temporal responses after temperature shift. Thus, temperature elevation induces a transient antimicrobial state in LL-37. The secondary structure of LL-37 rapidly reverted to its structure at 37 °C after being transferred from 40 °C to 37 °C for 10 min (Supplementary Fig. 2a), suggesting that the memory effect of antimicrobial activity from elevated temperature is not due to secondary structure changes. Instead, repeated crosslinking experiments consistently showed a modest increase in LL-37 oligomers following 40 °C incubation compared to 37 °C (Supplementary Fig. 2b), indicating that temperature-induced changes in LL-37 oligomerization might contribute to the memory effect, as the disassembly kinetics are significantly slower than conformational relaxation. In addition, the assembly could be further stabilized by bacterial membrane lipid^40^ or could be metastable, which could also contribute to the memory effect. Similarly, preincubation of histones or LL-37/histones dual treatment altered their membrane-permeabilizing activities (Fig. 2d, e). However, histones’ secondary structures have a diminished dependence on temperature compared to that of LL-37 (Supplementary Fig. 2c and Fig. 2a). Thus, changes in secondary structure and oligomerization of LL-37 provide a plausible mechanism for the increased antimicrobial activity of LL-37 at elevated temperature. We next wondered whether this increase was a consequence of reaction kinetics or a more subtle evolutionary tuning mechanism.

### AMP activity matches host temperature

**Fig. 3:**
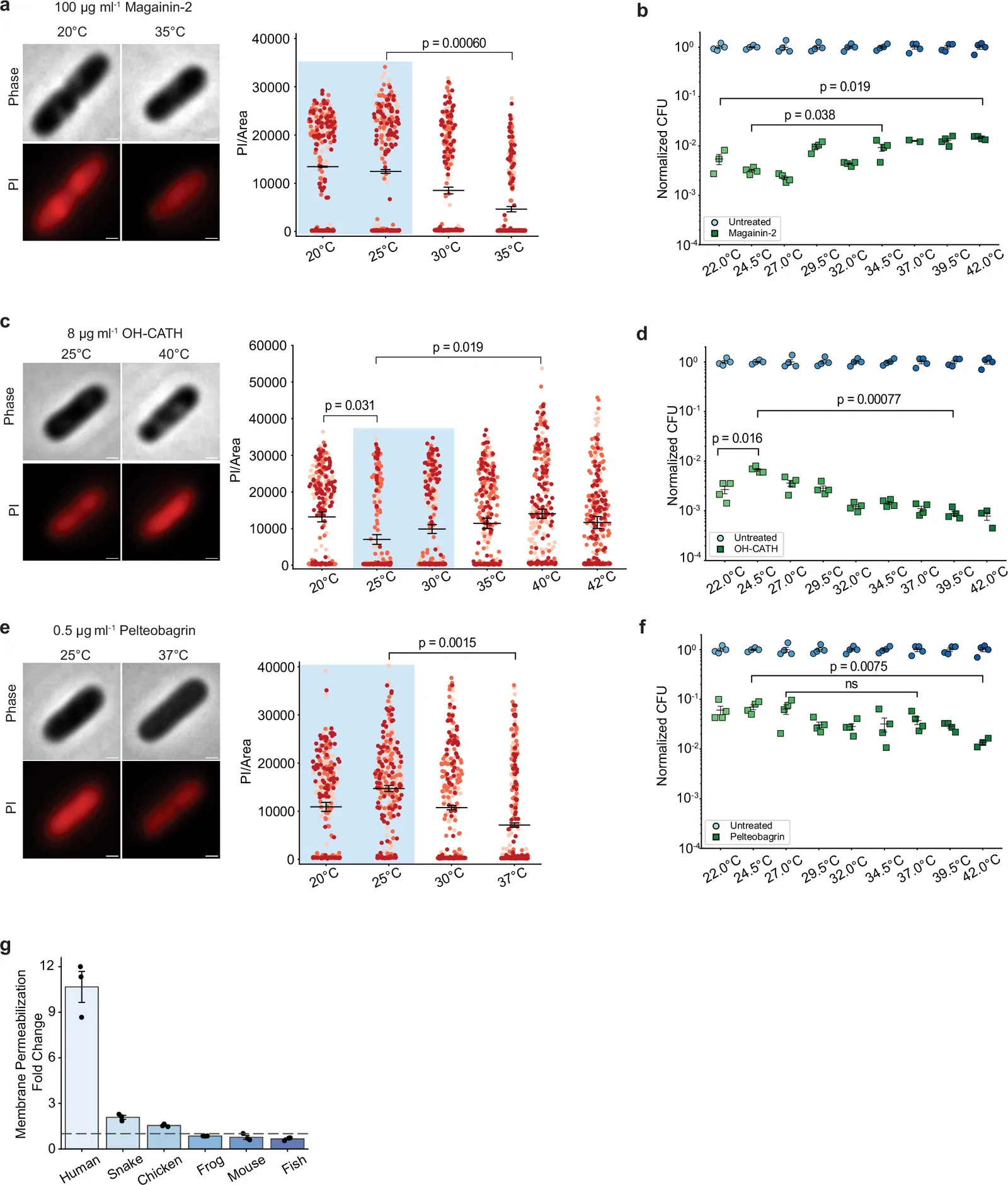
The antimicrobial activity of AMPs is optimized to host body temperature. **a** Representative phase-contrast and fluorescence images of PI entry for *E. coli* treated with 100 μg ml^−^^1^ magainin-2 (from the African clawed frog) for 1 h in MinA+ medium. PI fluorescence is quantified and plotted for each cell. **b** CFU assays following 1 h treatment with 5.4 μg ml^−^^1^ magainin-2 in water. **c** Representative images and quantifications following 1 h treatment with 8 μg ml^−^^1^ OH-CATH (from king cobra) in MinA+ medium. **d** CFU assays following 1 h treatment with 2 μg ml^−^^1^ OH-CATH in water. **e** Representative images and quantifications following 1 h treatment with 0.5 μg ml^−^^1^ pelteobagrin (from catfish) in water. **f** CFU assays following 1 h treatment with 10 μg ml^−^^1^ pelteobagrin in water. **g** Quantification of fold increase in the number of cells with PI entry at an elevated temperature compared to the animals’ optimal living temperature. Fold increase calculated using 5 μg ml^−^^1^ histones + 10 μg ml^−^^1^ LL-37 at 40 °C versus 37 °C for human; 8 μg ml^−^^1^ OH-CATH at 40 °C versus 25 °C for snake; 5 μg ml^−^^1^ CATH-1 for chicken at 43 °C versus 40 °C; 100 μg ml^−^^1^ magainin-2 at 30 °C versus 25 °C for frog; 100 μg ml^−^^1^ CRAMP at 40 °C versus 37 °C for mouse; 0.5 μg ml^−^^1^ pelteobagrin at 30 °C versus 25 °C for fish. The dashed line indicates an efficacy factor of 1. For CFU assays, three biological replicates were performed for Magainin-2 at 22 °C and OH-CATH and pelteobagrin at 42 °C. Four biological replicates for other conditions. Treatment groups were normalized to the untreated control at the corresponding temperature; data points indicate independent experiments. For PI assays, data points indicate individual cells. At least 100 cells were imaged per replicate; three biological replicates were performed for each condition and plotted as different colors. Black bars indicate the mean of independent experiments and error bars represent SEM. Scale bars represent 0.5 μm. Two-sided Welch’s t-tests with unequal variances were performed. No adjustments were made for multiple comparisons. *P*-values greater than 0.05 are denoted as non-significant (ns).

To determine whether the increase in LL-37 activity was simply a consequence of temperature-enhanced reaction kinetics, we looked at the activity of three other AMPs from species that have lower body temperatures: magainin-2 (frog), OH-CATH (king cobra snake), and pelteobagrin (catfish). We reasoned that if AMP activity was tuned to match body temperature, in these cases it would be maximized at a lower temperature. In contrast, if activity were determined by generic reaction kinetics, it should increase as the temperature increased. In most cases, we found that membrane-permeabilizing and bactericidal activities matched the typical body temperature ranges of the animals. For instance, the African clawed frog AMP magainin-2 was most active at 20 °C (Fig. 3a, b and Table 1), with its activity falling monotonically as temperature increased, consistent with the frog’s preference for cooler environmental temperatures^41^. In contrast, the snake’s AMP was active over a wide range of temperatures, from 20 °C to 40 °C (Fig. 3c, d and Table 1). This is consistent with the snake’s exposure to cool (~20 °C) temperatures at night, and warmer (30–35 °C) temperatures during the day^42^. Finally, the AMP from the yellow catfish shows the highest membrane permeability around 25 °C (Fig. 3e and Table 1), a typical ambient temperature for this fish^43^, with a decrease at 37 °C. We note that the CFU assay shows a slight increase in bactericidal activity with increasing temperature and does not follow the trends of the other AMPs (Fig. 3f). This difference likely reflects multiple killing mechanisms that have different temperature sensitivities (see discussion).

**Table 1:** Percentage of PI-positive *E. coli* cells following treatments with different peptides or proteins at different temperatures

Treatments	Temperatures	PI positive (%)
5 μg ml^−1^ histones + 5 μg ml^−1^ LL-37	37 °C	5.33%
	40 °C	23.76%
400 μg ml^−1^ H1	37 °C	14.67%
	40 °C	15.00%
10 μg ml^−1^ H1 + 2 μg ml^−1^ LL-37	37 °C	1.33%
	40 °C	18.00%
20 μg ml^−1^ LL-37	37 °C	17.69%
	38.5 °C	23.67%
	40 °C	28.90%
100 μg ml^−1^ histones	37 °C	34.67%
	38.5 °C	40.33%
	40 °C	57.33%
5 μg ml^−1^ histones + 10 μg ml^−1^ LL-37	37 °C	3.03%
	38.5 °C	18.52%
	40 °C	32.32%
0.5 μg ml^−1^ Pelteobagrin	20 °C	60.00%
	25 °C	84.67%
	30 °C	56.00%
	37 °C	41.33%
100 μg ml^−1^ Magainin^−^2	20 °C	64.33%
	25 °C	54.73%
	30 °C	42.18%
	35 °C	29.00%
8 μg ml^−1^ OH-CATH	20 °C	60.00%
	25 °C	32.00%
	30 °C	46.67%
	35 °C	56.58%
	40 °C	66.67%
	42 °C	62.67%
40 μg ml^−1^ LL-37	37 °C	35.00%
	40 °C	68.49%
	43 °C	69.88%
5 μg ml^−1^ CATH-1	37 °C	21.00%
	40 °C	43.33%
	43 °C	66.67%
100 μg ml^−1^ CRAMP	20 °C	3.96%
	34 °C	29.70%
	37 °C	63.37%
	40 °C	46.53%

Cells with an average PI/area greater than 1000 were classified as PI-positive.

We have already noted that the LL-37/histone combination showed increasing antimicrobial activity at elevated body temperatures, such as those reached during fever. Snakes, when infected, often bask on hot rocks in the sun to raise their body temperature^44^. The snake AMP’s membrane-permeabilizing activity at 40 °C, a temperature slightly above their expected daytime body temperatures, was significantly more active, following the increased membrane-permeabilizing trend of the human AMP, though not nearly as extreme (Fig. 3g). Thus, snakes appear to utilize approaches from both cold-blooded and warm-blooded evolution. We found no such increase in efficacy of the fish or frog AMPs at slightly higher than normal host temperatures (Fig. 3g), consistent with their lack of ability to increase their body temperatures.

Mice have an irregular regulation of body temperature in response to infection, with both increases^45,46^ and decreases^47,48^ in temperature observed. Interestingly, we observed that membrane permeabilization efficiency dropped in both directions moving away from their normal body temperature of 37 °C (Supplementary Fig. 3a), suggesting bacterial clearing is already at an optimum at normal body temperature. Like humans, chickens run a fever when infected^49^, however, their normal body temperature is around 41 °C^50^, and their fever temperature is around 43 °C^49^, both significantly higher than that of humans. One might therefore expect a shift in their AMPs’ effective temperature, and this is what was observed: in contrast to human cathelicidin, which has the same activity at 40 °C and 43 °C (Supplementary Fig. 3b), the chicken cathelicidin’s activity greatly increases (Supplementary Fig. 3c).

### Sequence variability between cathelicidins

To gain insight into the molecular determinants of the temperature response, we aligned the sequence of the four cathelicidins used in our experiments. While most residues are relatively well-conserved, there are key changes at specific residues that could alter temperature sensitivity. Compared to LL-37, all of the molecules have significant alterations at residues F5, K10, K15, R19, and D26 and in the last 3 to 5 residues of the C-terminus (Supplementary Fig. 3d, e). In addition, the first three hydrophobic residues are absent in mouse CRAMP, which has lower membrane permeabilizing activity than LL-37 in the shift from 37 °C to 40 °C. Chicken CATH−1, which has the highest peak efficacy at 43 °C, is missing residues K12 to K18 (corresponding to approximately 2 helix turns) and is the most hydrophobic cathelicidin tested. Finally, snake OH-CATH, which has two peak efficiencies, is missing residues R19-V21 but has a 3-residue insertion following N30.

Since organismal bacterial exposure can happen at any time, one might naively expect that the most powerful antibacterial defense would occur at normal body temperatures, yet for humans, snakes, and chickens, this does not appear to be the case. This raised an interesting possibility of a potential trade-off at elevated temperatures, and we wondered what it was.

### Temperature increases AMP and histone damage to mitochondria

Both AMPs and histones localize to cytosolic lipid droplets (LD)^23,51^, which provide fatty acids to mitochondria as a source for fatty acid metabolism^52,53^. Elevated AMP concentrations on LDs during infection might be detrimental because mitochondria have similar membrane physiology to bacteria^54,55^. AMPs permeabilize mitochondrial membranes and trigger apoptosis of mammalian cells^56^. We hypothesize that the temperature dependence of AMPs and histones reflects attempts to moderate this “off target” effect. That is, AMP activity is evolutionarily tuned to be moderate under regular conditions to protect mitochondria, but enhanced during fever when needed to expeditiously kill bacteria.

**Fig. 4:**
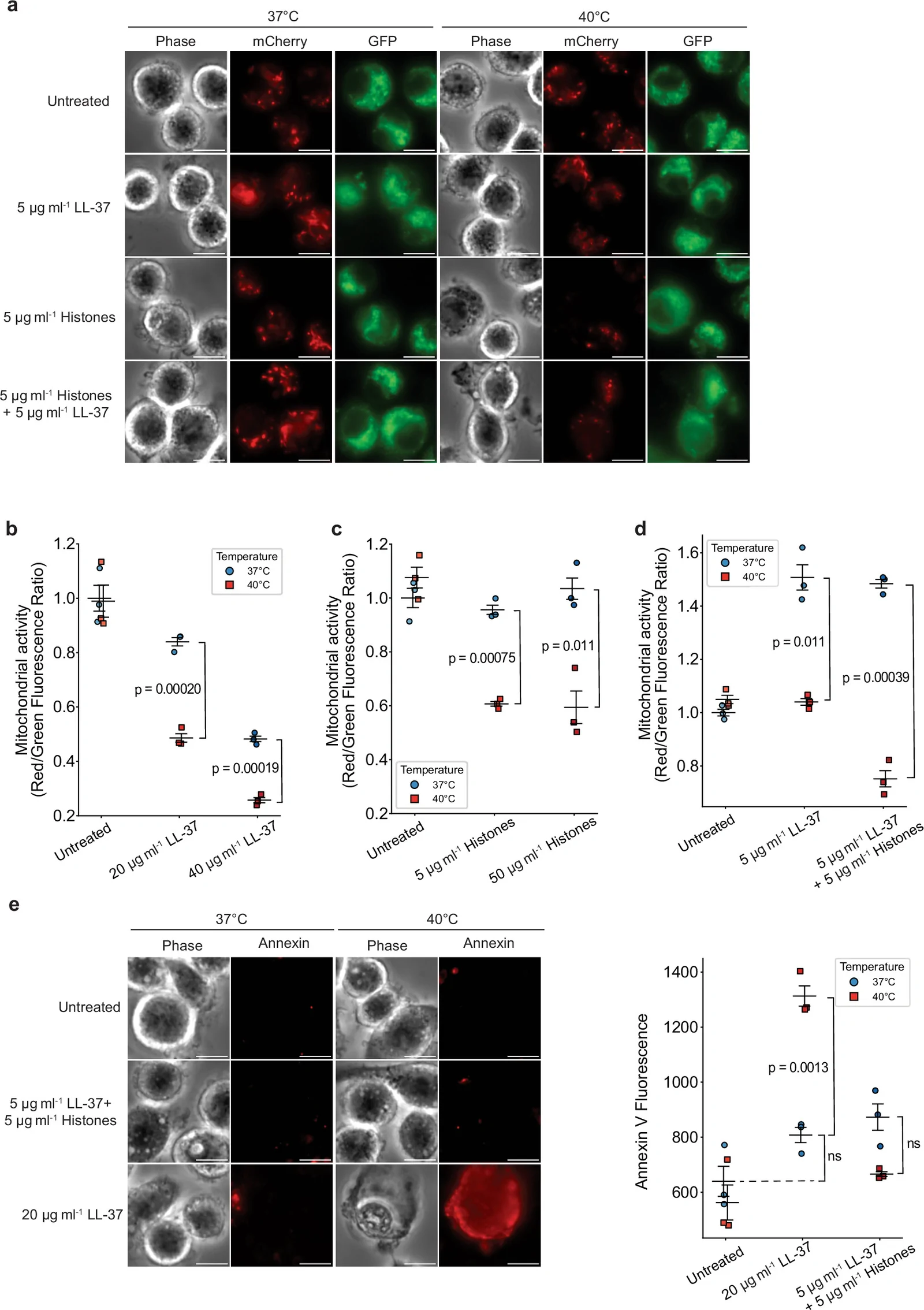
The synergistic antimicrobial activity of histones and LL-37 at febrile temperatures reduces apoptosis. **a** Representative fluorescence microscopy images of J774.1 macrophages following treatment with 5 μg ml^−^^1^ histones, 5 μg ml^−^^1^ LL-37, and the combined treatment. **b**-**d** Quantification of JC-1 red/green fluorescence ratio in macrophages treated with LL-37 (**b**), histones (**c**), or the combined treatment (**d**). Decreases in red/green ratios indicate membrane potential changes that are associated with mitochondrial damage. **e** Representative images and quantifications of Annexin V staining as an indicator of apoptosis. All treatments were performed for 1 hour. Data points represent the average of each independent experiment, for which at least 100 cells were imaged. Three biological replicates were performed for all conditions. Black bars indicate the mean of independent experiments, and error bars represent the SEM. The scale bar represents 10 μm. Two-sided Welch’s t-tests with unequal variances were performed. No adjustments were made for multiple comparisons. *P*-values greater than 0.05 are denoted as non-significant (ns).

In such a model, cells might be insensitive to small or moderate mitochondrial damage at normal body temperatures when pathogens are absent but then tolerant to more significant damage as a worthwhile trade-off when pathogens are present. This would be achieved if AMP and histone activity were temperature-dependent, and then raising the temperature in response to pathogens—i.e., the fever response. To test this possibility, we cultured macrophage J774.1 cells at 37 °C and exposed them to an exogenous supply of AMPs and histones in the surrounding medium. Because histones and AMPs can cross the eukaryotic cell membrane barrier^57–60^, this external treatment resulted in internal AMPs and histones, with some co-localization between AMPs, histones, and mitochondria (Supplementary Fig. 4a), allowing them to potentially affect mitochondrial function. We measured mitochondrial health using the JC−1 dye, which shows red fluorescence under high mitochondrial membrane potential and green fluorescence with depolarized mitochondria. Higher red/green ratios are thus associated with mitochondrial health. LL-37 and histones indeed entered cells and caused a dose-dependent decrease in mitochondrial health (Fig. 4a–d). When we raised the temperature to 40 °C, mitochondrial damage due to histone, LL-37, and dual LL-37/histone treatments was further increased (Fig. 4a–d). When we compared the mitochondrial damage due to the dual LL-37/histone treatment (5 μg ml^−1^ LL-37 + 5 μg ml^−1^ histones) with damage caused by LL-37 at a concentration (20 μg ml^−1^ LL-37) that has the same membrane permeabilization efficacy at 40 °C (Supplementary Fig. 4b, c), we found that the dual treatment (Fig. 4d) caused less mitochondrial damage than LL-37 alone (Fig. 4b).

Temperature can affect membrane permeability, so the increased mitochondrial damage might be due to increased AMP/histone entry rather than due to the AMP/histone temperature-dependent activity. Since there are free extracellular AMPs and histones in the body, this would still be biologically relevant, but we wanted to understand whether the AMP/histone temperature sensitivity was indeed important. Thus, we performed the mitochondrial damage assay using OH-CATH, which is less temperature sensitive. We found a modest increase in mitochondrial damage (around 1.2-fold; Supplementary Fig. 4d), which is consistent with its small PI increase (around 1.2-fold; Table 1) at elevated temperature. In contrast, LL-37 shows a greater temperature-dependent increase in both mitochondrial damage and PI uptake (around 1.7-fold increases for each at 37 °C vs. 40 °C using 20 μg ml^−1^ LL-37; Fig. 4b and Table 1). Although the measurements were performed using different probes and cannot be directly compared quantitatively, the trend with different peptides suggests that the mitochondrial damage is largely due to the temperature-sensitive nature of AMP activity rather than solely from a generic increase in membrane permeability or peptide uptake.

### Antimicrobial synergy reduces AMP-induced apoptosis

Since mitochondrial damage can trigger apoptosis^61,62^, we wondered whether the LL-37- or LL-37/histone-induced damage could lead to cellular apoptosis. To test this, we used the apoptosis dye Annexin V, which binds to phosphatidylserine that flips to the outer membrane leaflet during early apoptosis. We found that treating cells with a moderate level of external LL-37 at 37 °C did not increase apoptosis, but that a switch to 40 °C did (Fig. 4e). Further, as culturing the cells at 40 °C did not increase apoptosis unless LL-37 was added extracellularly, the increase in apoptosis can be attributed to mitochondrial damage by LL-37. Notably, when LL-37/histone are supplied at concentrations that give the same membrane permeabilization as LL-37 alone (Supplementary Fig. 4b, c), the dual LL-37/histone treatment caused much less apoptosis compared to LL-37 alone (Fig. 4e). The untreated and dual-treatment groups were statistically similar at both 37 °C and 40 °C, in contrast to the LL-37 treatment, where apoptosis was significantly increased at 40 °C. This suggests that one advantage of the synergistic effects of LL-37 and histone over LL-37 alone is that the synergistic activity is specifically tailored to affect bacteria, therefore reducing collateral mitochondrial damage.

### Temperature reduces LD-mitochondrial contact

Interestingly, an increase in temperature alone (without LL-37 or histone treatment) did not impair mitochondria (Fig. 4b, untreated). This was initially surprising to us, since even without exogenously supplying LL-37 and histones, both molecules are already present on lipid droplets (LDs)^23^, and we would expect LD-mitochondrial interactions to result in contact between mitochondria, LL-37, and histones, which would damage the mitochondria at the higher temperatures due to increased LL-37/histone activity. In our past work, we found that the body recognized infection and responded by increasing the concentration of histones and LL-37 on the LDs^23^. One of the corollaries of that increase was a concomitant decrease in LD-mitochondrial contacts^23^, presumably to protect the mitochondria. Indeed, in addition to our work here, past studies suggest that histones destabilize mitochondrial membranes^60^. Thus, we wondered whether the mitochondrial insensitivity to temperature could be due to the same mechanism—a decrease in LD-mitochondrial contact, achieved by increasing the LD-mitochondrial distance at higher temperatures.

**Fig. 5:**
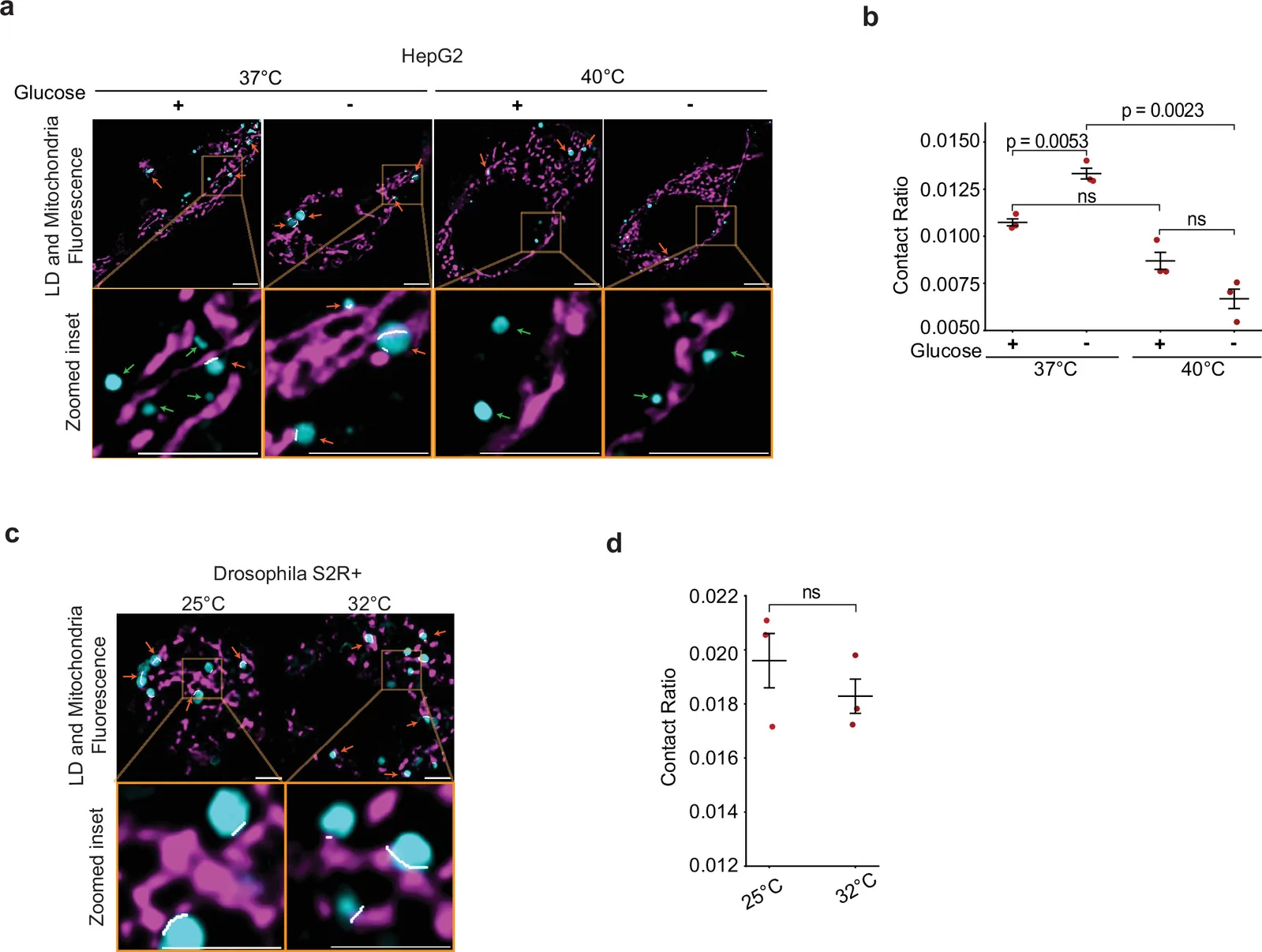
Lipid droplet-mitochondrial contacts as a function of temperature. **a** Representative structured illumination microscopy (SIM) images of mitochondria (magenta) and lipid droplets (cyan) in human hepatocytes HepG2 cells cultured with or without 4.5 g/L glucose. White lines indicate the contact regions. Green arrows indicate LDs without mitochondrial contact; orange arrows indicate contacted LDs. **b** Quantification of lipid droplet-mitochondria contact area in HepG2 from three biological replicates. **c** Representative SIM images of mitochondria (magenta) and lipid droplets (cyan) in *Drosophila* S2R+ cells. **d** Lipid droplet-mitochondria contact area for S2R+ cells. Contact ratios were computed by normalizing the total contact area by the total lipid droplet volume. Three biological replicates were done for each condition. Data points represent the average contact ratio from independent experiments, for which at least 50 cells were imaged. Black bars indicate the mean of independent experiments, and error bars represent SEM from three replicates. Scale bars represent 2 μm. Two-sided Welch’s t-tests with unequal variances were performed. No adjustments were made for multiple comparisons. *P*-values greater than 0.05 are considered non-significant (ns).

We assessed LD-mitochondrial contact using human hepatocyte (liver) HepG2 cells since hepatocytes play an important role in innate immunity^23^ and the flat morphology of HepG2 cells yields better imaging quality. First, we measured contacts at 37 °C under two different metabolic states: in a high-glucose state when cells were utilizing glucose and LD-mitochondrial interactions were expected to be minimal^63^, and a no-glucose state when LDs were docked to mitochondria, so that they could be actively used to provide fatty acids for fatty acid metabolism. As expected, we found that at 37 °C, the average LD-mitochondrial contacts in cells were significantly greater in the no-glucose state than in the high-glucose state (Fig. 5a, b). We next looked at LD-mitochondrial contact in a no-glucose state at 40 °C, and found that LD-mitochondrial contact ratios were much greater at 37 °C (Fig. 5a, b), suggesting minimal LD-mitochondrial interactions at 40 °C in a no-glucose environment. We also tested human lung epithelial A549 cells and observed a similar trend (Supplementary Fig. 5a, b), suggesting LD-mitochondrial separation may be generalized to other human cells. Thus, it appears that at high temperatures, LDs move away from mitochondria to avoid undue mitochondrial damage. As discussed below, this may explain why human metabolism is known to be altered at higher temperatures.

To investigate the mechanism of mitochondrial LD separation, we examined PLIN5, which is an LD surface protein that promotes LD-mitochondrial contacts. However, we found no significant decrease in PLIN5 association with LDs at 40 °C compared with 37 °C (Supplementary Fig. 5c, d), suggesting that this separation is likely regulated by mechanisms other than PLIN5. Since cold-blooded animals have chosen a different evolutionary route with less temperature-dependent AMP activity, one might expect that the LD-mitochondrial contacts would be less temperature-dependent in cells from cold-blooded animals. To test this, we quantified LD-mitochondrial contacts in *Drosophila* S2R+ cells at 25 °C vs 32 °C (going higher resulted in decreased cell viability in culture), and indeed observed no difference in contacts (Fig. 5c, d).

### AMP damage is evolutionarily tuned

**Fig. 6:**
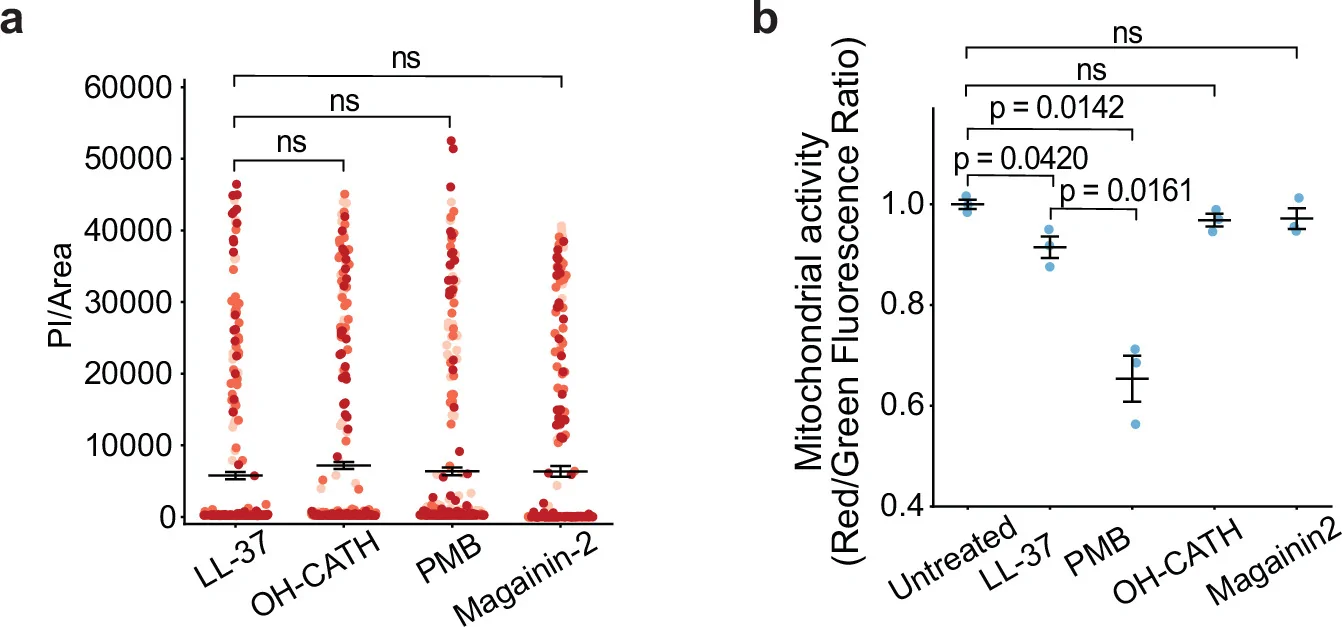
Cold-blooded animal AMPs are tuned to reduce mitochondrial damage. **a** Quantification of PI per cell following a 1-hour treatment at 37 °C of 20 μg ml^-1^ LL-37, 3 μg ml ^−1^ OH-CATH, 1 µg ml^−^^1^ PMB, and 100 μg ml^−^^1^ magainin-2. Three biological replicates were performed, and 100 cells were imaged in each replicate. **b** Quantification of mitochondrial activity of macrophages J774.1 using JC-1 red/green fluorescence ratio under the same conditions at 37 °C for 1 hour. Three biological replicates were performed, and at least 100 cells were imaged in each replicate. Black bars indicate the mean of independent experiments, and error bars represent the SEM. Two-sided Welch’s t-tests with unequal variances were performed. No adjustments were made for multiple comparisons. *P*-values greater than 0.05 are denoted as non-significant (ns).

Our observations are consistent with the general hypothesis that the fever response is protective: it dramatically increases antibacterial killing activity when needed, while still protecting mitochondria via a combination of moderate AMP activity at body temperatures, together with increased separation of LD-anchored AMPs from mitochondria at high temperatures where AMPs are more active. However, it raises an interesting question: how do non-mammalian animals—which cannot raise their body temperature—have effective antimicrobial activity while limiting damage to mitochondria? We initially considered that perhaps AMPs and histones were stored somewhere other than LDs, to prevent mitochondrial exposure, but that seems unlikely since there are high levels of histones on *Drosophila* LDs^51^. Instead, given that AMP activity—at least as far as temperature—can be selectively tuned evolutionarily, we wondered if AMP activity might be further tuneable as far as selectivity. That is, might it be possible to tune AMPs so that they increase bacterial damage relative to mitochondrial damage? To test this idea, we first looked at PMB, a last-resort antibiotic which is an AMP that is produced by the bacterium *Paenibacillus polymyxa* to fight other bacteria, and presumably has no evolutionary pressure to avoid mitochondrial damage. When we chose a PMB concentration to match bacterial damage done by LL-37 (Fig. 6a) and then tested both PMB and LL-37 on cells to assess mitochondrial impact, we found that PMB did significantly more mitochondrial damage (Fig. 6b). This was our first suggestion that it indeed might be possible to evolutionarily tune selectivity to bacteria vs mitochondria. With this in mind, we next did the same experiment to compare LL-37 activity to OH-CATH (from the king cobra) and magainin-2. We found that with similar membrane permeabilization activity at 37 °C (Fig. 6a), both OH-CATH and magainin-2 caused no significant mitochondrial damage, whereas LL-37 did (Fig. 6b). Thus, it appears that it is indeed possible to tune relative antibacterial vs anti-mitochondrial activity, and our data are consistent with the hypothesis that non-mammalian animals such as snakes and frogs have used this route to avoid mitochondrial damage. We did not include pelteobagrin in the above study because it is not active in the same medium as the others, so there was no clean way to do such a comparison. These findings were consistent with the lack of change in LD-mitochondrial contacts as a function of temperature observed in *Drosophila* S2R+ cells, presented above.

## Discussion

The mechanisms that improve clearing of bacteria through fever have not been understood. Here, we have found that the antimicrobial activity of AMPs and histones increases significantly with temperature. At the heart of this mechanism is the histone/AMP synergism. The activity of both LL-37 and histones is temperature-dependent, and their synergistic combination achieves multiplicative antimicrobial activity and thus takes better advantage of small changes in temperature, allowing for a more dramatic increase in bacterial killing efficacy compared to LL-37 alone. While the antimicrobial activity of LL-37 rises with temperature, there is a significant trade-off for the host: LL-37 significantly increases damage to the mitochondria (Fig. 4b). However, for a fixed level of membrane permeabilization activity, the combination of histones and LL-37 causes less mitochondrial damage and less apoptosis than LL-37 alone. Synergy that arises from histones and LL-37 thus has the added benefit of increasing antimicrobial activity while mitigating mitochondrial damage. Furthermore, it may be harder for bacteria to evolve resistance to the multi-pronged attack^64,65^, as this likely requires independent evolution along multiple axes.

AMPs have the highest activity near the typical body temperature of the animal from which they come. For animals that are able to raise their body temperature, the maximal antimicrobial activity occurs at slightly elevated temperatures. This is not an accident, as the AMPs from animals unable to raise their body temperatures do not show this activity. Our data thus suggest that AMP activity reflects an evolutionary trade-off: because of their lipid droplet-associated nature^23,51^, AMP antimicrobial activity also damages mitochondria; thus, for mammals, AMP activity at normal body temperature must be kept moderate. When invading microorganisms are detected, the body temperature is increased, maximizing AMP activity and activating synergy with histones.

Our findings show that human cells avoid excessive damage from LL-37 at elevated temperatures by moving LDs away from mitochondria, while the *Drosophila* cells do not. The protection of mitochondria through distancing has an unexpected impact on metabolism: as LDs move away from mitochondria, the latter’s access to fatty acids is restricted, and so glycolysis is the best option. This interpretation addresses an open issue: why does heat induce a shift towards glycolysis and away from fatty acid metabolism^66–68^? While it has been suggested that the impairment is due to protein unfolding^69^, our results suggest this could be attributed to LD-mitochondrial distancing instead in order to avoid the damaging effects of AMPs.

For non-mammalian animals, our data suggest a different path: instead of tuning AMP activity to be strongly temperature sensitive, and then using the fever response to kill bacteria, non-mammalians likely used evolutionary tuning of AMP activity to decrease mitochondrial damage while maintaining the ability to kill bacteria. Snakes fall into an interesting intermediate zone, where they have minimized AMP-related mitochondrial damage but retain moderate temperature sensitivity, potentially to take advantage of basking in the sun. We speculate that this moderate increase in efficacy reflects the difficulty of satisfying these two evolutionary constraints. One obvious prediction from our work is that metabolism of non-mammalian animals should be much less temperature sensitive, consistent with the observation that in *Drosophila*, temperature does not appear to affect LD-mitochondrial interactions. Overall advantages of the different strategies remain to be explored in future work; however, from a practical point of view, we suggest that for antibacterial drug development, it might be prudent to start with AMPs from non-mammalian animal sources, as they are likely to have less off-target effects (mitochondrial damage) when applied systematically, as compared to bacterially derived AMPs such as PMB, or mammalian AMPs that may be optimized for temperature dependence rather than decreased mitochondrial damage. We also note the interesting case of the pelteobagrin AMP, which may be worth further study: while its membrane permeabilization activity (judged by PI entry) is consistent with the AMPs from other cold-blooded animals unable to change their body temperature, the observed killing activity (measured by CFUs) increases with temperature, at higher temperatures than expected to be relevant for the catfish (Fig. 3e, f). Our previous analysis of AMP activity^27^, indicates that AMPs and histones likely have dual mechanisms of action, with one resulting from membrane permeabilization, and the other reflecting internal bacterial damage due (e.g.) to altering DNA structure/accessibility. The divergence between PI entry and killing suggests that pelteobagrin may exhibit a dual mechanism that’s different from the other AMPs. Understanding this mechanism could prove illuminating for future AMP development.

Our data may help rationalize the relatively recent observation that, on average, human body temperatures have decreased over the last hundred years^70,71^. Above, we suggested that the AMP’s activity was likely the result of an evolutionary compromise between maximizing bacterial killing efficacy and minimizing mitochondrial damage. Over the last hundred years, as sanitary conditions have improved, our day-to-day exposure to bacterial infections has decreased. Thus, it may be possible to re-evaluate the trade-off, and accept slightly worse bacterial protection in exchange for better mitochondrial health. The simplest way to achieve this seems to be to lower the body temperature, thus lowering the AMPs' efficacy and their associated damage to mitochondria. Indeed, our data suggest that even a few degrees decrease in temperature can materially decrease mitochondrial damage. Specifically, LL-37 treatment that decreases mitochondrial activity by 0.6-fold at 37 °C has no effect at 35 °C (Supplementary Fig. 5e, f).

Finally, our data suggest that inhibiting fever could impair the body’s efficacy in fighting bacterial infections. Antipyretic drugs are commonly used to reduce moderate fevers during infection, though this could have the unintended effect of reducing antimicrobial activity. Indeed, even moderate fever (38.5 °C) is associated with significant improvement in bacterial killing (Fig. 1 and Supplementary Fig. 1b and Table 1). The extent to which these matter in a clinical setting remains to be explored more fully, but our data suggest that rather than decreasing body temperature, perhaps local heating at the sites of infection should be more actively employed. With the dramatic increase in antibiotic-resistant bacteria, a deeper understanding of how the body fights infection becomes increasingly important.

## Methods

### Bacterial growth conditions and reagents

*E. coli* MG1655^72^ was streaked on Petri dishes with LB-Miller (BD Biosciences, Franklin Lakes, NJ) and 2% Bacto agar (BD Biosciences). Plates were incubated at 37 °C overnight to obtain single colonies. Single colonies were cultured into MinA medium (4.5 g KH_2_PO_4_, 10.5 g K_2_HPO_4_, 1 g (NH_4_)_2_SO_4_, and 0.5 g sodium citrate • 2H_2_O per 1 L water) supplemented with 1 mM MgSO_4_, 0.2% glucose and 0.1% casamino acids^27^. The complete medium is hereafter referred to as MinA+ medium. The cultures were grown overnight at 37 °C on a roller drum at 18 rpm, then diluted 1:100 into fresh medium and grown until OD_600_ reached 0.2 to 0.4. Then exponential-phase cells were diluted to an OD_600_ of 0.04 into fresh medium before treatment.

Antimicrobial agents, including bovine histone H1 (Sigma-Aldrich), calf thymus unfractionated type II-A whole histones (Sigma-Aldrich, St. Louis, MO), human cathelicidin LL-37 (Anaspec, Fremont, CA), polymyxin B sulfate (Sigma-Aldrich), and chicken CATH-1 (NovoPro Bioscience, Shanghai, China**)**, were prepared in 0.1 M sodium bicarbonate (pH 8.3) and stored at −20 °C. OH-CATH (NovoPro Bioscience), pelteobagrin (NovoPro Bioscience), magainin-2 (AnaSpec, Fremont, CA) and mouse CRAMP (AnaSpec) were prepared in water and aliquots were stored at −80 °C.

Histones and LL-37 were fluorescently labeled using Atto 488 NHS Ester (Sigma-Aldrich) by mixing the molecules with the dye at a molar ratio of 1:2 in 0.1 M NaHCO_3_. The mixed solution was placed on a shaking platform for 1 h in the dark at room temperature. Unreacted dye was removed using PD MidiTrap G-25 columns (GE Healthcare Life Sciences, Pittsburgh, PA). The labeled solutions were then aliquoted and stored at −80 °C. Each aliquot was thawed only once before use. The final Atto 488-labeled histones or LL-37 solution contains 10% labeled and 90% unlabeled molecules.

### Cell lines and growth conditions

Human hepatocytes HepG2 cells (ATCC HB-8065), human A549 cells (ATCC CCL-185), and mouse J774A.1 macrophages (ATCC TIB-67) were cultured in high-glucose DMEM (Gibco) supplemented with 10% fetal bovine serum (HyClone, Logan, UT) and 1% penicillin-streptomycin (Life Technologies) in a 37 °C humidified incubator with 5% CO_2_. For LL-37 or histone treatment, J774.1 cells were incubated with the indicated molecules at 37 °C or 40 °C for 1 h. J774.1 cells were passaged using a cell scraper; HepG2 cells were passaged using TrypLE (Gibco) for 7 min, and A549 cells were passaged using 0.05% trypsin/0.53 mM EDTA in DPBS (Gibco) for 5 min. At least three passages were done before the experiment. For no-glucose treatment, cells were transferred to glucose-free DMEM (Gibco) supplemented with 1% penicillin-streptomycin overnight before the experiment and incubated at 37 °C or 40 °C for 1 h before measurement.

*Drosophila* S2R+ cells (DGRC Stock 150; RRID: CVCL_Z831) were cultured in Schneider’s Drosophila Medium (Thermo Fisher Scientific, Waltham, MA) supplemented with 10% fetal bovine serum (HyClone) in 25 °C incubator with ambient CO_2_. Cells were passaged every two days by dilution into fresh medium. For temperature treatment, cells were incubated at either 25 °C or 32 °C for 1 h.

### Epifluorescence microscopy

We used a Nikon Eclipse Ti-E microscope (Nikon, Melville, NY) containing a 100X Plan Apo (1.45 N.A.) objective, a Sola light engine (Lumencor, Beaverton, OR), an LED-DA/FI/TX filter set (Semrock, Rochester, NY) containing a 409/493/596 dichroic and a Hamamatsu Orca Flash 4.0 V2 camera (Hamamatsu, Bridgewater, NJ). For propidium iodide (PI) fluorescence, 10 μL of bacterial culture was added to slides with 1% agarose before imaging. 575/25 nm excitation, 641/75 nm emission filters, with laser power 25% and 100 ms exposure time were used. LUT is linear and covers the full range of the data.

For mitochondrial membrane potential, J774.1 mouse macrophages were plated into a 60 × 15 mm Falcon Petri dish with a cover glass coated with 0.1% (w/v) poly-L-lysine (Sigma-Aldrich) on top 24 h before the experiment. Treatment was directly added to the Petri dish for 1 h. And JC-1 membrane potential detection kit (Biotium) was used to stain cells for 20 min at 37 °C, washed twice with PBS, pH 7.4 (Thermo Fisher Scientific). Microchambers were made using cover glass attached to slides by double-sided tape and imaged immediately. We used the 575/25 nm excitation and 641/75 nm emission filters for the red channel and 474/27 nm excitation and 525/45 nm emission filters for the green channel. Both channels were imaged using 50% laser power and 200 ms exposure time. Fold changes of mitochondrial damage are calculated using red/green fluorescence relative to the stated baseline. For apoptosis imaging, Annexin V Apoptosis Detection Kits (Thermo Fisher Scientific) were used to stain the cells for 15 min at 37 °C, then washed twice with PBS and assembled into a microchamber before imaging. 575/25 nm excitation and 641/75 nm emission filters were used for imaging apoptosis. An exposure time of 200 ms with laser power 50% was used. LUT is linear and covers the full range of the data. All images were obtained using Nikon NIS-Elements version 4.5 and analyzed using AVIassembleGUI^73^ version 1.3 in MATLAB R2023a (MathWorks, Natick, MA) (see ‘Code Availability’ section).

### Immunofluorescence

HepG2 cells were plated in 35-mm i-bioTreat µ-Dishes (ibidi, Fitchburg, USA) 24 h before the experiment. Cells were exposed to 37 °C or 40 °C for an hour before fixation. Cells were fixed with 4% formaldehyde for 15 min, permeabilized with 0.2% Triton X-100 (Sigma-Aldrich) for 5 min, and blocked using 5% BSA (Sigma-Aldrich) for 1 h at room temperature. Cells were then incubated overnight at 4 °C with primary antibody anti-Perilipin 5 (C-terminus) guinea pig polyclonal (ProGen, Cat. No. GP31) diluted 1:200 in 1% BSA. The primary antibody was detected with Alexa Fluor 647-conjugated goat anti-guinea pig IgG (H + L) highly cross-adsorbed secondary antibody (Thermo Fisher Scientific, Cat. No. A-21450) diluted 1:500 in 1% BSA for 1 h at room temperature. Then lipid droplets were stained with 1 μg ml^−^^1^ BODIPY (Thermo Fisher Scientific) for 20 min in DPBS. Cells were washed three times using DPBS between each step.

### Lattice SIM^2^ microscopy

For mitochondrial and lipid droplet imaging, cells were plated in 35-mm ibiTreat µ-Dishes (ibidi, Fitchburg, USA) 24 h before the experiment. Cells were incubated at 37 °C or 40 °C for 1 h. Before imaging, mitochondria were stained with 0.055 μg ml^−^^1^ Mitotracker (Thermo Fisher Scientific), and lipid droplets were stained by 1 μg ml^−^^1^ BODIPY (Thermo Fisher Scientific) for 20 min and imaged immediately. Super-resolution images were acquired using Elyra 7 Lattice Structured Illumination Microscope (SIM) (Carl Zeiss, White Plains, NY) equipped with a 63x/1.4 Oil Plan-Apo objective and dual camera: 2 pco.edge 4.2 camera system. Z-stacks were performed with a 0.329 μm step interval using ZEN Black (version 3.0). Live-cell imaging was performed under 5% CO_2_ at either 37 °C or 40 °C. Images were acquired using 561 nm laser for MitoTracker, 488 nm for BODIPY and Atto 488-labeled histones or LL-37, and 642 nm for PLIN5 immunostaining. An exposure time of 50 ms was consistently used for all channels. Laser power was set to 0.2% for BODIPY, 0.3% for Mitotracker, PLIN5 and Atto 488-labeled histones or LL-37. Raw SIM images were reconstructed using Zen Black edition (version 3.0) with SIM^2^, 3D Leap mode, regularization weight of 0.065, and iteration 20. LUT is linear and covers the full range of the data. For contact area analysis, we developed an ImageJ (2.16.0/1.54p) macro that can batch process two-channel 3D SIM images in a memory-efficient way. Each channel was processed using a Gaussian blur (σ = 5 pixels), background subtraction (rolling ball radius = 50 pixels), and Otsu dark thresholding. The resulting masks were used to calculate contact area between two channels. The contact area was normalized by the total volume of lipid droplets and reported as contact ratio (see ‘Code Availability’ section).

### Circular dichroism

Circular dichroism (CD) spectrum was collected using a JASCO J-810 spectropolarimeter (Jasco International Co. Ltd., Hachioji City, Japan) with a temperature controller. Jasco Spectra Manager (version 2.13) was used for instrument control and data acquisition. LL-37 or histones were diluted in MinA salt medium to a final concentration of 0.125 mg ml^−1^ and 0.1 mg ml^−1^, respectively. Samples were loaded into a 1 mm path length quartz cuvette (Starna Scientific, Atascadero, CA). Far-UV CD spectra were measured from 190 nm–260 nm at indicated temperatures with a scan speed of 100 nm/min, a bandwidth of 1 nm and 3 accumulations per measurement. At least four replicates were performed for each sample. Samples were equilibrated to the target temperature for 15 min before measurement. The α-helical and antiparallel β-sheet contents were estimated using BeStSel^38^ (last updated 18 March 2024), then the mean and SEM were calculated across all replicates in each condition.

### Glutaraldehyde crosslinking

LL-37 was diluted in MinA medium to 1 mg ml^−1^ and incubated in a 37 °C or 40 °C water bath for 1 h. Glutaraldehyde working solution was freshly prepared from a 25% glutaraldehyde stock solution (Sigma-Aldrich) by dilution in deionized water. LL-37 samples were mixed with glutaraldehyde to final concentrations of 0.01% immediately after temperature treatment and incubated in 37 °C for 5 min for crosslinking. Reactions were quenched by adding 1 M glycine. Samples were then loaded onto 16.5% Mini-PROTEAN Tris-Tricine gels (Bio-Rad, Hercules, CA) and analyzed by SDS-PAGE. A Spectra Multicolor Low Range Protein Ladder (Thermo Scientific, Chino, CA) was used as a molecular weight marker.

### Membrane permeability assays

Membrane permeability was visualized by propidium iodide (PI) fluorescence. Mid-exponential cells were diluted to an OD_600_ of 0.04 in fresh MinA+ medium and treated with antimicrobial peptides. Chicken CATH-1 treatments were performed for 20 min at the indicated temperatures to prevent complete cell lysis, whereas treatments with all other peptides were performed for 1 h. Peptide preincubation experiments were performed in a water bath and followed by adding peptides to bacterial cultures for 10 min. All peptide treatments were performed in MinA+ medium except for pelteobagrin, which was tested in water by centrifuging and resuspending the cells, as the antimicrobial effect is highly sensitive to salt concentration^74^. Then cells were supplemented with 8 μM PI for 10 min at 37 °C and imaged immediately.

### CFU assays

Overnight *E. coli* cells were grown to mid-exponential phase in MinA+ medium, diluted 1:20 into fresh medium, then treated with antimicrobial peptides, histones, or dual treatment for 1 h at indicated temperatures on block heaters. After treatment, cultures were diluted in ten-fold serial dilutions from 10^0^ to 10^6^ into water, 10 µl droplets were plated onto non-selective LB-Miller agar plates, incubated for 18 h at 37 °C, and counted for single colonies. For treatment, histones and LL-37 were performed in MinA+ medium, whereas other peptides were tested in water.

### Peptide sequence analysis and visualizations

Cathelicidin sequences were obtained from the official vendors’ websites. Multiple-sequence alignment was performed using MUSCLE 5^75^ and visualized in Jalview (v2.11.5.2) using the Clustal color scheme. Net charge and hydrophobicity were calculated using the Database of Antimicrobial Activity and Structure of Peptides (DBAASP)^76^. LL-37 structure was obtained from the RCSB Protein Data Bank (PDB ID: 2K6O^77^), and structures of the other peptides were predicted using AlphaFold^78^. All structures were visualized in UCSF ChimeraX (v1.12).

### Statistical analyses

Two-sided Welch’s t-tests with unequal variances for all data were performed with Python 3.10.9 using scipy.stats v.1.10.0 (SciPy, scipy.org). Two-way ANOVAs were performed with Python 3.10.9 using statsmodels.formula.api.ols v.0.13.5 (statsmodels, statsmodels.org).

### Reporting summary

Further information on research design is available in the Nature Portfolio Reporting Summary linked to this article.

## Supplementary information

Supplementary Information

Reporting Summary

Transparent Peer Review file

## Source data

Source Data

## Data Availability

The structured illumination microscopy images for HepG2 cells and uncropped protein gel images from the crosslinking experiment are available in Zenodo at https://doi.org/10.5281/zenodo.22262306. All data needed to evaluate the conclusions in the paper are present in the paper and/or the Supplementary Information. Raw data for all figures, including statistical analyses, are available in Source Data. Additional raw data or materials used in this study are available upon request. Correspondence and requests for materials should be addressed to asirya@uci.edu or sgross@uci.edu. Source data are provided with this paper.

## Code availability

The custom ImageJ macro used for mitochondria-LD contact analysis is available on Zenodo at https://doi.org/10.5281/zenodo.22262306^79^. The custom MATLAB code^73^ used for PI fluorescence, JC−1, and Annexin V Apoptosis fluorescence quantification is available on Zenodo at https://doi.org/10.5281/zenodo.15200252^80^.
