# Supplementary material for: Fever enhances host bacterial defence while limiting mitochondrial damage
Source: Nat Commun. 2026 Oct 14;17:11618. doi: 10.1038/s41467-026-78498-6 (PMC13754849; doi:10.1038/s41467-026-78498-6)
Supplement: Supplementary file 1 [file 41467_2026_78498_MOESM1_ESM.pdf]

SUPPLEMENTARY INFORMATION

Fever enhances host bacterial defence while limiting mitochondrial damage

Yonghan Wu, Elijah Rowe, Albert Siryaporn, Steven P. Gross

Corresponding author: asirya@uci.edu or sgross@uci.edu.

Contents:

Supplementary Figures 1-5

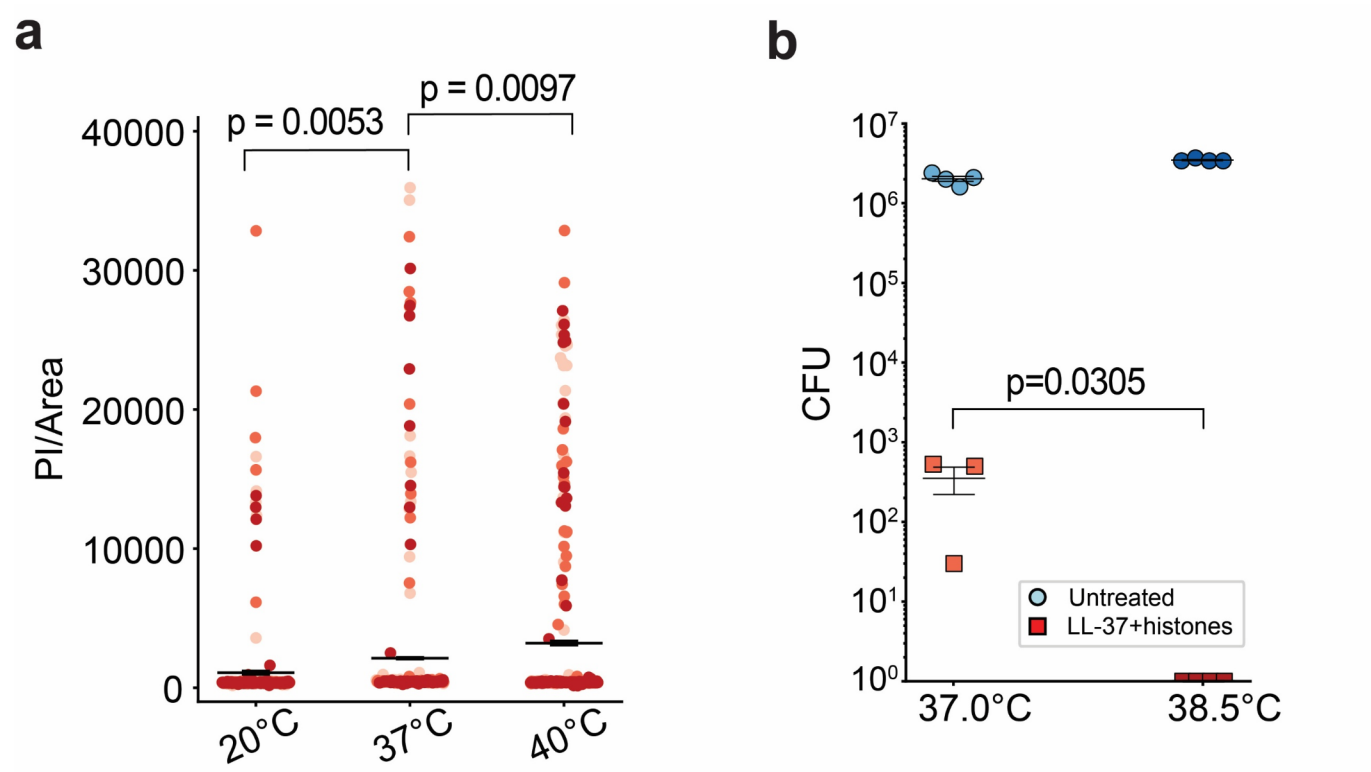

Supplementary Fig. 1. Quantification of propidium iodide (PI) fluorescence intensity and CFU at different treatments.

(a) PI quantification for *E. coli* MG1655 treated with 100 μg ml^{-1} LL-37 in DMEM cell culture medium with 10% fetal bovine serum at indicated temperatures for 1 h. (b) CFUs performed at indicated temperatures for 5 μg ml^{-1} whole histone and 20 μg ml^{-1} LL-37 dual treatment for 4 h. For the PI experiment, three biological replicates were done for each condition and plotted as different color densities. 100 cells were imaged in each replicate. The average fluorescence of each cell is illustrated as a dot in the plot. Black bars indicate the mean and error bars represent standard error of the mean (SEM) from three replicates. Scale bar = 0.5 μm. Two-sided Welch's t-tests with unequal variances were performed. No adjustments were made for multiple comparisons. P-values greater than 0.05 are denoted as non-significant (ns).

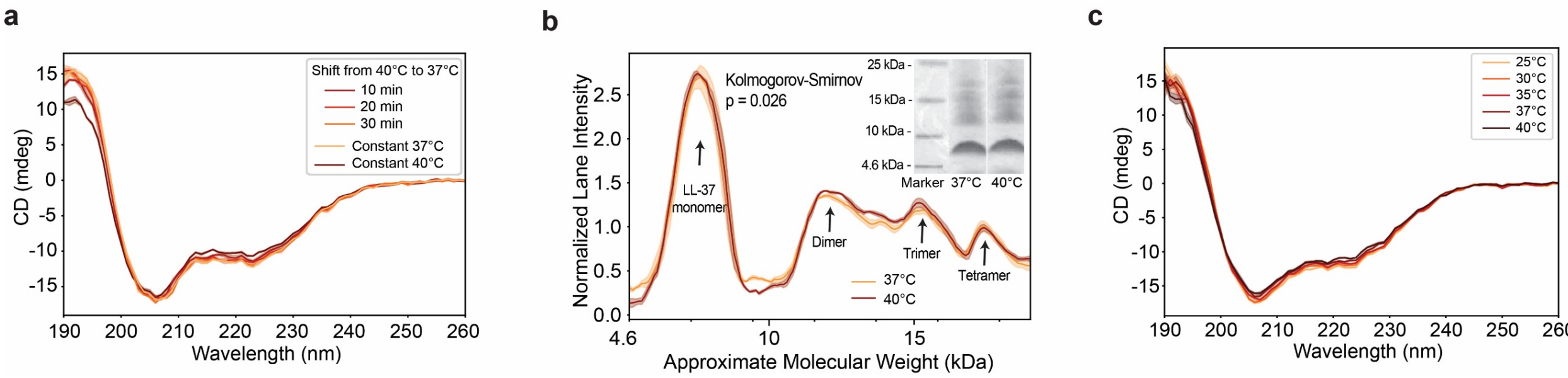

Supplementary Fig. 2. Circular dichroism (CD) spectra and crosslinking analyses of LL-37 and histones at different temperatures.

(a) CD spectra for 0.125 mg ml^{-1} LL-37 in MinA medium equilibrated at 40°C for 1 h and then shifted to temperature 37°C for the indicated times. Spectra at 37°C and 40°C are included for comparison. Six technical replicates were performed at 40°C, three at each time point (10 min, 20 min and 30 min) and five at 40°C. (b) Glutaraldehyde crosslinking gel analysis of 1 mg ml^{-1} LL-37 in MinA medium following 1 h incubation at 37°C or 40°C. Band intensity profile was plotted from bottom to top of the gel. Three independent replicates were performed and mean with SEM was plotted. Intensity of each lane was normalized by total lane signal. Kolmogorov-Smirnov test was performed between to curve with p = 0.0264. Original gel scan and other replicates are provided in Source Data file. (c) CD spectra for 0.1 mg ml^{-1} histones (containing core and linker histones) in MinA medium under different temperatures. Each CD spectrum is the average of four technical replicates for 25°C, 30°C and 35°C, five replicates for 37°C and six for 40°C. Solid lines represent the mean and shaded regions show the SEM.

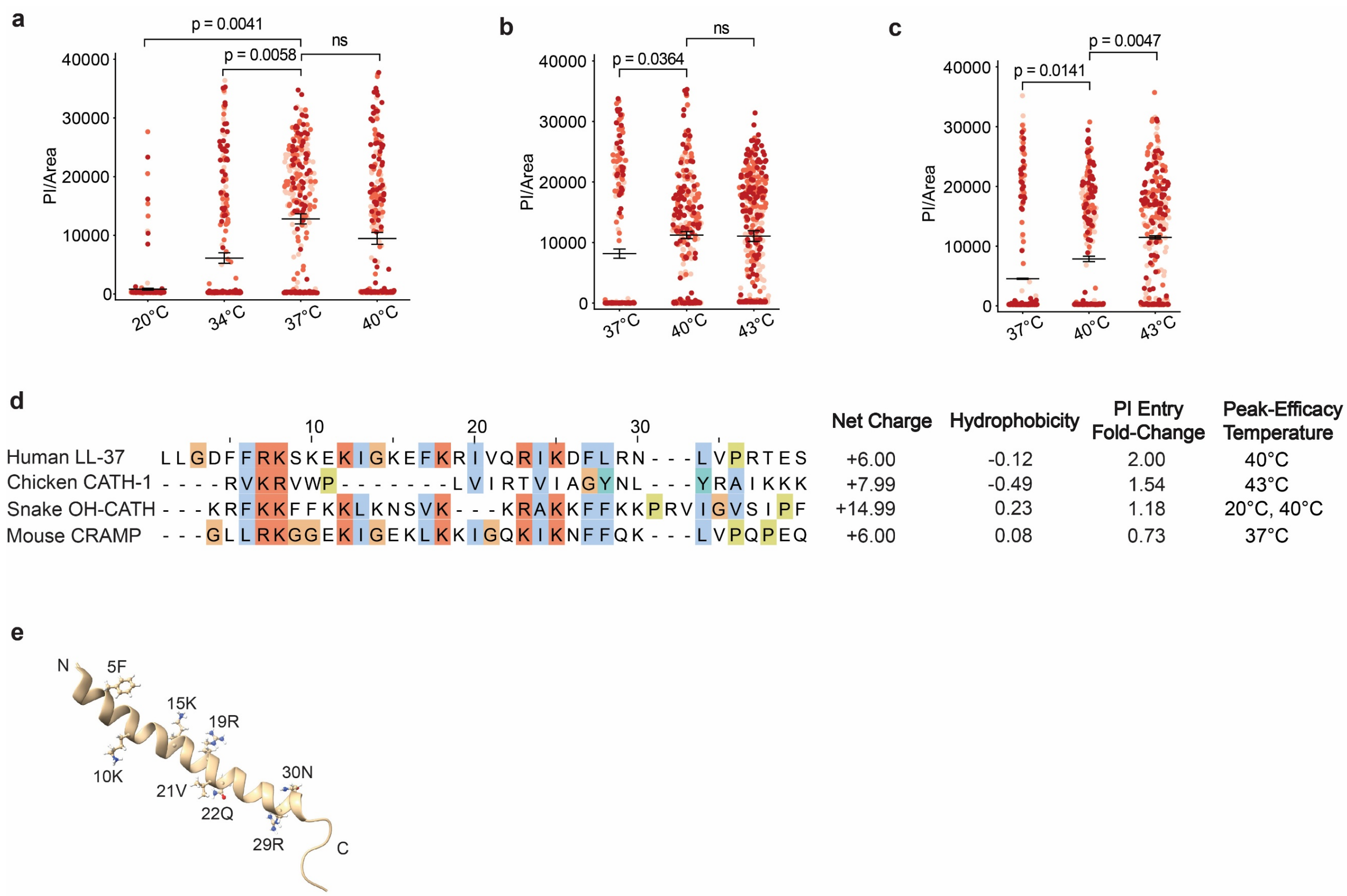

Supplementary Fig. 3. Membrane permeability activity and sequence alignment of cathelicidins from different host.

(a-c) PI fluorescence for *E. coli* MG1655 treated with (a) 100 µg ml^{-1} CRAMP for 1 h, (b) 40 µg ml^{-1} LL-37 for 1 h and (c) 5 µg ml^{-1} chicken CATH-1 for 20 min at the indicated temperatures. All treatments were performed in MinA+ medium. Three biological replicates were performed for each condition and plotted as different color densities. 100 cells were imaged in each replicate. Black bar indicated the mean and error bars represented SEM from three replicates. Two-sided Welch's t-tests with unequal variances were performed for all comparisons. No adjustments were made for multiple comparisons. P-values greater than 0.05 are denoted as non-significant (ns). (d) Multiple-sequence alignment of human LL-37, chicken CATH-1, snake OH-CATH, and mouse CRAMP. Sequences were aligned using MUSCLE5 and visualized using Clustal color scheme. Net charge and normalized Moon and Fleming scale hydrophobicity were determined using Database of Antimicrobial Activity and Structure of Peptides (DBAASP). PI entry fold-changes were calculated using PI data in Table 1: 40°C vs. 37°C for LL-37, CATH-1 and CRAMP, and 40°C vs. 35°C for OH-CATH. Peak-efficacy temperatures were also obtained from Table 1. (e) LL-37 structure obtained from RCSB Protein Data Bank (PDB ID: 2K6O) with residues 5,10,15,19,21,22,29,30 visible as sticks.

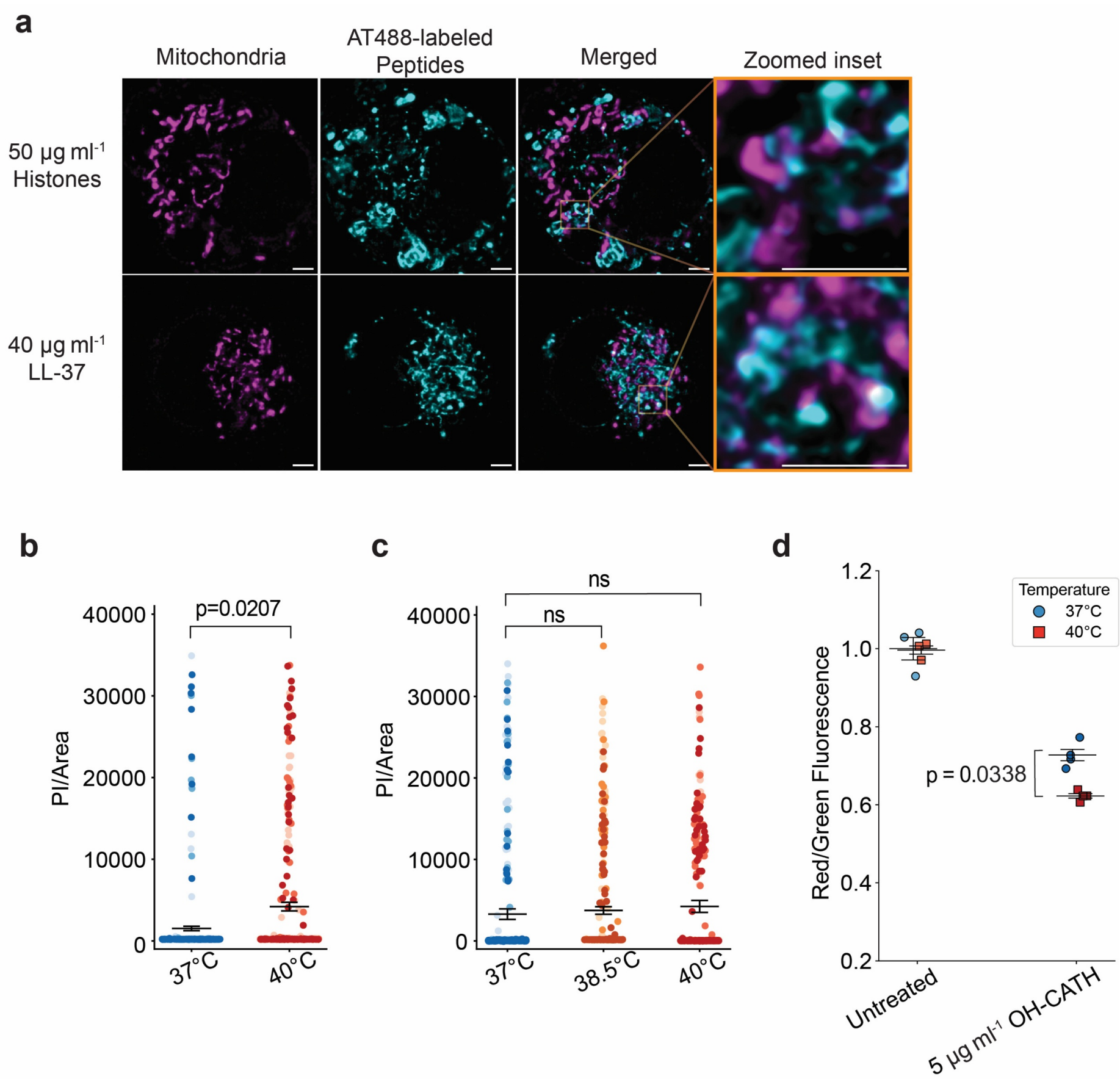

Supplementary Fig. 4. Antimicrobial localization, PI and mitochondrial activity following different treatment at physiological or febrile temperatures.

(a) Localization of 40 μg ml^{-1} LL-37 (cyan) or 50 μg ml^{-1} histones (cyan) within macrophages and their proximity to mitochondria (magenta) at 37°C, imaged using structured illumination microscopy (SIM). Three biological replicates were performed for each condition. At least 50 cells were imaged in each replicate. Scale bar represents 2 μm. (b,c) PI quantification for *E. coli* MG1655 treated with 5 μg ml^{-1} histones and 5 μg ml^{-1} LL-37 (b), and 20 μg ml^{-1} LL-37 (c) at indicated temperatures for 1 h. (d) Quantification of JC-1 red/green fluorescence ratio of 5 μg ml^{-1} OH-CATH treated J774.1 for 1 h at 37°C and 40°C. Three biological replicates were done for each condition. At least 100 cells were imaged in each replicate. Black bar indicated the mean and error bars represented SEM from three replicates. Two-sided Welch's t-tests with unequal variances were performed. No adjustments were made for multiple comparisons. P-values greater than 0.05 are denoted as non-significant (ns).

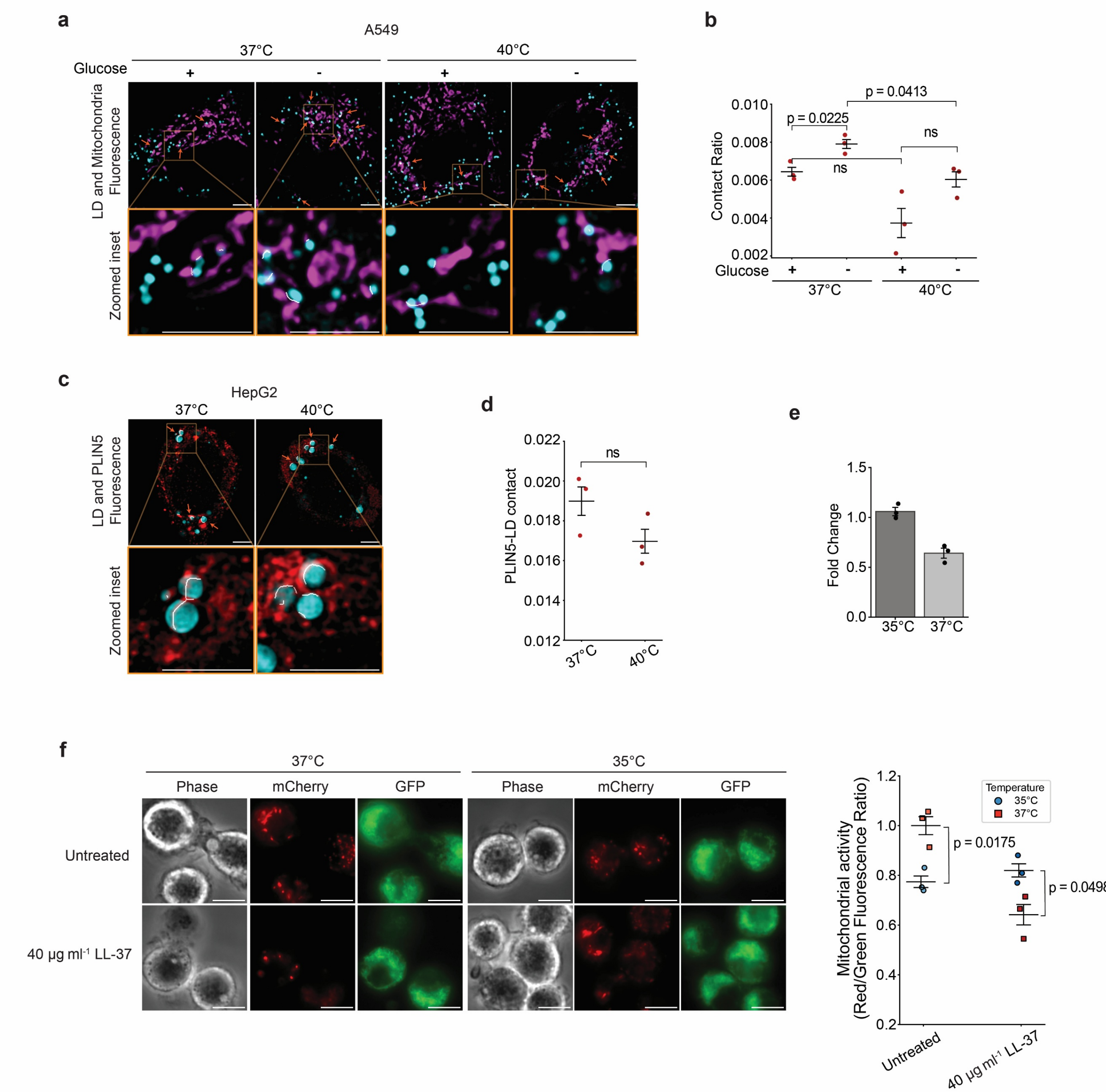

Supplementary Fig. 5. Mitochondrial activity and mitochondria-LD contacts in response to antimicrobial treatment and temperature.
(a) Representative SIM images of mitochondria (magenta) and lipid droplets (cyan) in human epithelial A549 cells incubated in 4.5 g/L glucose or no glucose medium for 12 h, with the final 1 h at 37°C or 40°C. White lines indicate the contact regions. Scale bar represents 4 µm. (b) Quantification of mitochondria-lipid droplet contact of A549 cells, calculated by the total contact area normalized to the total lipid droplet volume. (c) Representative SIM images for human HepG2 cells incubated in no glucose medium for 12 h, with the final 1 h at 37°C or 40°C. PLIN5 staining is shown in red and lipid droplets in cyan. White lines indicate the contact regions. Scale bar represents 4 µm. (d) Quantification of PLIN5-lipid droplet contact, calculated by the total contact area normalized to the total lipid droplet volume. Three biological replicates were done for each condition. At least 50 cells were imaged in each replicate. (e) Fold-changes of

mitochondrial activity for 40 μg ml^{-1} LL-37 normalized by untreated at 35°C and 37°C. (f) Representative fluorescence microscopy images of J774.1 macrophages with 1 h treatment of 40 μg ml^{-1} LL-37 at 37°C and 35°C and quantification of JC-1 red/green fluorescence ratio. Scale bar represents 10 μm. Black bar indicated the mean and error bars represented SEM from three replicates. Two-sided Welch's t-tests with unequal variances were performed. No adjustments were made for multiple comparisons. P-values greater than 0.05 are denoted as non-significant (ns).
