# Supplementary material for: Fever enhances host bacterial defence while limiting mitochondrial damage
Source: Nat Commun. 2026 Oct 14;17:11618. doi: 10.1038/s41467-026-78498-6 (PMC13754849; doi:10.1038/s41467-026-78498-6)
Supplement: Supplementary file 2 [file 41467_2026_78498_MOESM2_ESM.pdf]

nature portfolio

Corresponding author(s): Steven Gross, Albert Siryaporn

Last updated by author(s): Sep 4, 2026

# Reporting Summary

Nature Portfolio wishes to improve the reproducibility of the work that we publish. This form provides structure for consistency and transparency in reporting. For further information on Nature Portfolio policies, see our Editorial Policies and the Editorial Policy Checklist.

## Statistics

For all statistical analyses, confirm that the following items are present in the figure legend, table legend, main text, or Methods section.

| n/a | Confirmed | |
|---|---|---|
| ☐ | ☒ | The exact sample size (*n*) for each experimental group/condition, given as a discrete number and unit of measurement |
| ☐ | ☒ | A statement on whether measurements were taken from distinct samples or whether the same sample was measured repeatedly |
| ☐ | ☒ | The statistical test(s) used AND whether they are one- or two-sided<br>*Only common tests should be described solely by name; describe more complex techniques in the Methods section.* |
| ☒ | ☐ | A description of all covariates tested |
| ☐ | ☒ | A description of any assumptions or corrections, such as tests of normality and adjustment for multiple comparisons |
| ☐ | ☒ | A full description of the statistical parameters including central tendency (e.g. means) or other basic estimates (e.g. regression coefficient) AND variation (e.g. standard deviation) or associated estimates of uncertainty (e.g. confidence intervals) |
| ☐ | ☒ | For null hypothesis testing, the test statistic (e.g. *F*, *t*, *r*) with confidence intervals, effect sizes, degrees of freedom and *P* value noted<br>*Give P values as exact values whenever suitable.* |
| ☒ | ☐ | For Bayesian analysis, information on the choice of priors and Markov chain Monte Carlo settings |
| ☒ | ☐ | For hierarchical and complex designs, identification of the appropriate level for tests and full reporting of outcomes |
| ☒ | ☐ | Estimates of effect sizes (e.g. Cohen's *d*, Pearson's *r*), indicating how they were calculated |

*Our web collection on statistics for biologists contains articles on many of the points above.*

## Software and code

Policy information about availability of computer code

| | |
|---|---|
| Data collection | For propidium iodide, JC-1 and Annexin V Apoptosis data, phase-contrast and fluorescence images were taken using Nikon NIS-Elements (version 4.5). For Lattice SIM2 microscopy, ZEN Black (version 3.0) were used, and circular dichroism spectra were collected using Jasco Spectra Manager (version 2.13). |
| Data analysis | For propidium iodide, JC-1 and Annexin V Apoptosis fluorescence quantification, custom MATLAB code using Canny edge detection was used to identify cell boundaries from phase contrast images and then fluorescence intensities inside each mask were extracted for each cells. MATLAB r2023a was used. Custom MATLAB code is available on GitHub at [https://github.com/asirya/AVIassembleGUI] and in Zenodo at [https://doi.org/10.5281/zenodo.15200252]. For lipid droplet and mitochondria contact analysis, raw SIM images were first reconstructed using ZEN Black (version 3.0). Then use a custom ImageJ (version 1.54p) macro to quantify the contact areas. The macro applied Gaussian filtering and intensity thresholding to get LD and mitochondrial mask, then detect the overlapping regions to obtain contact masks to quantify contact area. The custom ImageJ macro is available at [https://github.com/asirya/MitoContactArea] and in Zenodo at at [https://doi.org/10.5281/zenodo.22262306]. Peptides' secondary structure contents were estimated using BeStSel (last updated 18th March, 2024). |

For manuscripts utilizing custom algorithms or software that are central to the research but not yet described in published literature, software must be made available to editors and reviewers. We strongly encourage code deposition in a community repository (e.g. GitHub). See the Nature Portfolio guidelines for submitting code & software for further information.

## Data

Policy information about availability of data

All manuscripts must include a data availability statement. This statement should provide the following information, where applicable:
- Accession codes, unique identifiers, or web links for publicly available datasets
- A description of any restrictions on data availability
- For clinical datasets or third party data, please ensure that the statement adheres to our policy

Dataset including HepG2 structured illumination microscopy images and uncropped protein gels are available at Zenodo at [https://doi.org/10.5281/zenodo.22262306]. Raw data for all figures including statistical analyses are available in Source Data file. Additional raw data supporting the findings of this study are available upon request at asirya@uci.edu or sgross@uci.edu.

## Research involving human participants, their data, or biological material

Policy information about studies with human participants or human data. See also policy information about sex, gender (identity/presentation), and sexual orientation and race, ethnicity and racism.

| | |
|---|---|
| Reporting on sex and gender | Not applicable |
| Reporting on race, ethnicity, or other socially relevant groupings | Not applicable |
| Population characteristics | Not applicable |
| Recruitment | Not applicable |
| Ethics oversight | Not applicable |

Note that full information on the approval of the study protocol must also be provided in the manuscript.

# Field-specific reporting

Please select the one below that is the best fit for your research. If you are not sure, read the appropriate sections before making your selection.

[x] Life sciences [ ] Behavioural & social sciences [ ] Ecological, evolutionary & environmental sciences

For a reference copy of the document with all sections, see nature.com/documents/nr-reporting-summary-flat.pdf

# Life sciences study design

All studies must disclose on these points even when the disclosure is negative.

| | |
|---|---|
| Sample size | At least three independent biological replicates were performed for each condition. For microscopy experiments, at least 100 cells were analyzed per biological replicate for bacteria and at least 50 cells were analyzed for eukaryotic cell. Sample sizes were evaluated to ensure they exceeded the minimum required to determine the statistical significance and reproducibility. Sample sizes are reported in the figure legends. |
| Data exclusions | No data were excluded. |
| Replication | All attempts at replication were successful. Experimental findings were verified using at least three independent biological replicates, indicated in the corresponding figure legends |
| Randomization | Randomization was not relevant to this study because samples were assigned to predefined experimental conditions and processed in parallel under identical conditions. |
| Blinding | Blinding was not performed to this study because data collection and analysis have used automated and objective methods, including batch processing of PI fluorescence and SIM imaging and CFU counting. |

# Reporting for specific materials, systems and methods

We require information from authors about some types of materials, experimental systems and methods used in many studies. Here, indicate whether each material, system or method listed is relevant to your study. If you are not sure if a list item applies to your research, read the appropriate section before selecting a response.

## Materials & experimental systems

| n/a | Involved in the study | |
|---|---|---|
| | x | Antibodies |
| | x | Eukaryotic cell lines |
| x | | Palaeontology and archaeology |
| x | | Animals and other organisms |
| x | | Clinical data |
| x | | Dual use research of concern |
| x | | Plants |

## Methods

| n/a | Involved in the study | |
|---|---|---|
| x | | ChIP-seq |
| x | | Flow cytometry |
| x | | MRI-based neuroimaging |

# Antibodies

| | |
|---|---|
| Antibodies used | Guinea pig polyclonal anti-Perilipin 5 (C-terminus; ProGen, Cat. No. GP31; 1:200 dilution) and Alexa Fluor 647-conjugated goat anti-guinea pig IgG (H+L), highly cross-adsorbed (Thermo Fisher Scientific, Cat. No. A-21450; 1:500 dilution). |
| Validation | Both Guinea pig polyclonal anti-Perilipin 5 antibody and Alexa Fluor 647-conjugated goat anti-guinea pig IgG (H+L) antibody were validated by the manufacturer using immunofluorescence and western blotting. |

# Eukaryotic cell lines

Policy information about cell lines and Sex and Gender in Research

| | |
|---|---|
| Cell line source(s) | J774A.1, A549, and HepG2 cells were purchased from ATCC (catalog no. TIB-67, CCL-185, and HB-8065, respectively). Drosophila S2R+ cells were obtained from the Drosophila Genomics Resource Center (DGRC Stock 150; RRID:CVCL_Z831). |
| Authentication | J774A.1, A549, and HepG2 cells were authenticated by ATCC before purchase. The identity of the Drosophila S2R+ cell line was verified by DGRC before purchase. |
| Mycoplasma contamination | HepG2 cells were tested for mycoplasma contamination every two weeks and remained negative throughout the study. J774A.1, A549, and Drosophila S2R+ cells were not tested for mycoplasma contamination. |
| Commonly misidentified lines (See ICLAC register) | No commonly misidentified cell lines listed in the ICLAC register were used in this study. |

# Plants

| | |
|---|---|
| Seed stocks | Not applicable |
| Novel plant genotypes | Not applicable |
| Authentication | Not applicable |
