# Supplementary material for: Fever enhances host bacterial defence while limiting mitochondrial damage
Source: Nat Commun. 2026 Oct 14;17:11618. doi: 10.1038/s41467-026-78498-6 (PMC13754849; doi:10.1038/s41467-026-78498-6)
Supplement: Supplementary file 3 [file 41467_2026_78498_MOESM3_ESM.pdf]

nature portfolio

Peer Review File

Fever enhances host bacterial defence while limiting mitochondrial damage

Corresponding Author: Professor Steven Gross

This file contains all reviewer reports in order by version, followed by all author rebuttals in order by version.

Version 0:

Reviewer comments:

Reviewer #1

(Remarks to the Author)
The study has characterised the role of temperature on the activity antimicrobial peptide LL-37 and histones, suggesting a protective role of increased temperature (fever) in the immune response. Interestingly, the increase in activity is not found with peptides from cold-blooded animals. The authors then investigate the effect of temperature on mitochondria and lipid droplets interactions, suggesting a protective mechanism in mammalian cells. Taken together, this is a well-performed study that reports interesting findings but is overestimating some of the data. More robust data would be needed.

Major comments

1. The first results on antimicrobial peptide activity does not take into account that temperature could affect bacteria as well. Previous publications have shown a variation of antimicrobial resistance upon culture temperature (Reinés et al, Journal of bacteriology, 2012). Another publication (Mackowiak et al, Journal of Infectious diseases, 1982) showed a lower resistance to antibiotics upon culture temperature. Thus, one can wonder if the antimicrobial effect (CFU and PI staining) is more due to a change in the bacteria (25c vs 40c) rather than a change in the activity of the peptide ?
One experiment could be to incubate the peptide at different temperatures and then incubate on bacteria at 37C ? This should be at least discussed and references maybe added.

2. Could it be possible to test the activity/structure of the peptide LL-37 and Histones upon temperature ? It is surprising that 3C (37 to 40) could change so much the activity of the peptides. Reference 13 (x et al) suggests that this change of activity is linked to change of structure.

3. The exploration of differences between species is really interesting. Chicken have a body temperature of 40C, would chicken Cathelicidin show more antibacterial activity ? Furthermore, it has been debated that mice have hypothermia instead of fever during infection. Would this go against the hypothesis of the work ? Would mice AMP more active at lower temperature ?

4. Page 10, line 154. The authors claim that LL37 and histones have entered the cells. It seems that there are no direct proof of this. The peptides could have as well triggered signalling pathways from the membrane of the macrophages. Furthermore, is there any indication that LL-37 or histone can binds to mitochondria ? - In addition to this point: How purified/clean are the antimicrobial peptides ? Endotoxin contamination could activate J774.1 macrophages and play a role on mitochondria activation/health.

5. The data on the contact points with LDs is interesting. The microscopy would benefit from a zoomed inset. If the authors' hypothesis are correct, using cold-bloaded animals cell line would not show such a unloading effect. Furthermore, if an active separation LD/mitochondria is happening, what is the mechanism ? Could this be driven by PLINs proteins ? Does heat-shock induce a switch in PLIN expression (PLIN5 to PLIN2 or vice-versa ?) ?

6. Finally, Is there any effect on mitochondrial dynamic (fission/fusion) between 37C and 40C ?

Minor comments:
7. P15: figure 5b is called in the text before Fig 5a

8. P9, line 148: I am guessing the macrophages were cultured at 37C and not at room temperature ?
9. P27, line 477: uM instead of microM
10. Figure 3 panel E is confusing. Cells have been treated with annexin (575/25 nm excitation and 641/75 nm) but the right graph show Red/Green Fluorescence (575/25 nm excitation and 641/75 nm emission filters for the red channel and 474/27 nm excitation and 525/45 nm emission filters). Are both Red emissions imaged together ? Please explain.
11. In general the image magnification is not high enough (figure 3 in particular)

Overall, the manuscript is well written and the basic premise of the study is fundamentally sound. Whilst the level of work can be appreciate, it lacks more robust data to really confirm the findings.

Reviewer #2

(Remarks to the Author)
The manuscript by Yonghan Wu et al. investigates the role of fever-range temperatures (37° C to 40°C) on the efficacy of the human antimicrobial peptide (AMP) LL-37. Using complementary approaches, the authors suggest that hyperthermia enhances LL-37 activity against pathogens without a proportional increase in mitochondrial damage. This provides a compelling evolutionary argument for fever as a host defense mechanism.
While the topic is novel and of high interest to a general audience, several experimental discrepancies and technical omissions must be addressed.

Major points

1) Peptide Aggregation and Conformational State: LL-37 is well-known to oligomerize and is highly prone to aggregation, which significantly impacts its biological activity (see Nature Communications 2020, 11:3859 and Biochem J. 2014, 457(3):451-61). Have the authors analyzed how temperature increases specifically alter the aggregation state or secondary structure of LL-37? Can the authors exclude the possibility that the observed "efficiency" is an artifact of experimental buffer conditions favouring a specific oligomeric state that might differ from the complex physiological environment of human macrophages ?

2) Inconsistencies in Imaging and Quantitation (PI Assay): The representative images for membrane permeabilization (Propidium Iodide entry) are not convincing and appear to contradict the quantified data:
Figure 1b vs. 1d: In Fig 1b (E. coli + LL-37 at 37°C), the fluorescence is invisible, yet the reported PI/Area is around 8,000-10,000. In contrast, Fig 1d (LL-37 + Histone) shows clearly visible fluorescence, yet the mean PI/Area is reported as only 5,000.
Figure 2a vs. 2b: For Pelteobagrin, a PI/Area of 6,000- 7,000 is reported for both panels, yet the signal is clearly visible in 2a and virtually absent in 2b (35°C).
The authors must provide truly representative images or explain these numerical discrepancies.

3. Discrepancies between Text and Data (Cold-blooded AMPs): There are some contradictions in the reporting of the antimicrobial activity (CFU counts) in Figure 2:
Pelteobagrin: The text and also fluorescence PI assay states activity decreases with temperature, but Figure 2e appears to show an significant increase in activity (CFU counts) as temperature rises.
Magainin: The data does not support a temperature-dependent decrease in activity. CFU counts only appear to change at 42°C, with no significant trend at intermediate (37°C) temperatures.
The authors need to carefully re-examine their figures and ensure the text accurately reflects the shown results.

4) Synergy Limitations: In the synergistic assays, only a single concentration combination was tested for the two molecules. The authors must justify how these specific concentrations were chosen and demonstrate their physiological relevance within a cellular environment.

5)Quantification of Mitochondrial Damage (Lines 160–166). The methodology used to calculate the fold increase in mitochondrial damage is not clearly described. The values provided (1.2-fold and 1.7-fold) appear to derive from two distinct experiments measuring different biological phenomena using different fluorescent probes. Absolute fold-change values can only be directly compared if a precise correlation exists between the underlying physiological phenomenon and the specific sensitivity/linear range of each probe.

6) Quantification of Annexin V (Figure 3): The authors must clarify the biological and physical significance of the 'Red/Green Ratio' reported in Figure 3. Annexin V and PI are independent markers for distinct cellular events (membrane inversion vs. nuclear membrane rupture). Therefore, a ratio between these two signals is unconventional and lacks a standardized scale.

Minor Points

Line 125: Define the specific baseline/control groups used to calculate the "fold increase" in PI-positive cells in Figure 2d.

Line 94: Note that OH-CATH is a cathelicidin like LL-37. Discussing their sequence differences could explain their divergent thermal performance.

Figure 1: State the incubation times for all antimicrobial assays; this is essential for interpreting membrane permeabilization kinetics.

Line 129: Please, standardize all peptide concentrations. Currently, Magainin is in µM while others are in µg/ml.

Version 1:

Reviewer comments:

Reviewer #1

(Remarks to the Author)
The authors have answered all my comments and have made their conclusion cleary more robust. This is a very nice piece of work that will hopefully lead to more interesting discoveries.
Congratulation on the authors for this nice work and their great effort to answer all queries from reviewers.

(Remarks on code availability)
I do not have the expertise to review the code

Reviewer #2

(Remarks to the Author)
In this revised manuscript, the authors explore how elevated temperature enhances the activity of human LL-37 and histones, proposing a physiological role for fever in mammalian host defense.

The authors have addressed several initial concerns by performing additional experiments, which are now included in the Supplementary Information. While these new additions clarify certain aspects, several important technical and presentation issues remain:

1.Figure Organization and Logical Flow:
Heavy reliance on Supplementary Figures—discussed with equal weight to main figures, forces the reader to constantly flip between documents. Reorganizing the main and supplementary figures is essential to consolidate related data within single panels, ensuring the authors' rationale and scientific narrative remain coherent without page-switching.

2.Biophysical Interpretation and Structural Data (Figures 2 & S2):

a) LL-37 CD Spectral Interpretation (Figure 2): The authors attribute increased activity at higher temperatures to a structural transition based on Far-UV CD spectra. However, the characteristic α-helical minima (208 nm and 222 nm do not shift position, and signal variation is minimal. Minor ellipticity changes without peak shifts typically reflect temperature-dependent solvent dynamics rather than secondary structure alterations.

b) Histone H1 CD Spectrum (Figure S2e): The reported CD spectrum for histone H1 appears characteristic of a fully alpha-helical protein. In free solution, full-length H1 is dominated by its disordered tails and exhibits a high random-coil content !. The authors should clarify the buffer conditions or construct used.

c) C) Inconsistent Oligomerization and "Memory Effect" (Figure S2b): No oligomerization differences are observed between 37° C and 40° C (supplementary Fig. S2b), which contradicts the claim that LL-37 retains a 45-minute "memory effect" after incubation at 40 ° C. Because conformational relaxation of a 37-residue peptide occurs on microsecond timescales, a 45-minute kinetic lag requires a plausible physical rationale (e.g., slow assembly/disassembly kinetics or metastable aggregation).

(Remarks on code availability)

Open Access This Peer Review File is licensed under a Creative Commons Attribution 4.0 International License, which permits use, sharing, adaptation, distribution and reproduction in any medium or format, as long as you give appropriate credit to the original author(s) and the source, provide a link to the Creative Commons license, and indicate if changes were made.
In cases where reviewers are anonymous, credit should be given to 'Anonymous Referee' and the source.
The images or other third party material in this Peer Review File are included in the article's Creative Commons license, unless indicated otherwise in a credit line to the material. If material is not included in the article's Creative Commons license and your intended use is not permitted by statutory regulation or exceeds the permitted use, you will need to obtain permission directly from the copyright holder.
To view a copy of this license, visit https://creativecommons.org/licenses/by/4.0/

Author responses in blue. Line numbers refer to main text without changes tracked (clean version).

Reviewer #1 (Remarks to the Author):
The study has characterised the role of temperature on the activity antimicrobial peptide LL-37 and histones, suggesting a protective role of increased temperature (fever) in the immune response. Interestingly, the increase in activity is not found with peptides from cold-blooded animals. The authors then investigate the effect of temperature on mitochondria and lipid droplets interactions, suggesting a protective mechanism in mammalian cells. Taken together, this is a well-performed study that reports interesting findings but is overestimating some of the data. More robust data would be needed.

We thank the reviewer for their evaluation. We have performed a number of new experiments and analyses that have increased the rigor and impact of the findings. We have fully addressed the reviewer's concerns below.

Major comments

1. The first results on antimicrobial peptide activity does not take into account that temperature could affect bacteria as well. Previous publications have shown a variation of antimicrobial resistance upon culture temperature (Reinés et al, Journal of bacteriology, 2012). Another publication (Mackowiak et al, Journal of Infectious diseases, 1982) showed a lower resistance to antibiotics upon culture temperature. Thus, one can wonder if the antimicrobial effect (CFU and PI staining) is more due to a change in the bacteria (25c vs 40c) rather than a change in the activity of the peptide ? One experiment could be to incubate the peptide at different temperatures and then incubate on bacteria at 37C ? This should be at least discussed and references maybe added.

We thank the reviewer for this suggestion. The fact that Magainin-2 and Fish AMPs don't do better at higher temperatures argues against a purely bacterial explanation. We have now directly done the experiments suggested by the reviewer. We incubated the peptide at 40°C and 37°C and then treated bacteria that were cultured at 37°C. Pre-incubation of LL-37 or the combination of histones with LL-37 at 40°C increased their membrane permeabilizing activity towards *E. coli* more than those pre-incubated at 37°C (Fig. 2b, Supplementary Fig. 2c, d). Furthermore, LL-37 showed a memory effect following its incubation at 40°C and its increased efficiency gradually decreased over time, returning to the activity of 37°C within 45 minutes (Fig. 2c). We have also added a citation to the previous study showing that growth temperature affects bacterial antimicrobial resistance.

2. Could it be possible to test the activity/structure of the peptide LL-37 and Histones upon temperature ? It is surprising that 3C (37 to 40) could change so much the activity of the peptides. Reference 13 (x et al) suggests that this change of activity is linked to change of structure.

We thank the reviewer for this suggestion. We have now characterized the structural response of LL-37 to temperature using circular dichroism and oligomerization assays. LL-37 shows a temperature-dependent change in secondary structure and also a slight change in oligomerization with increased temperature (Fig. 2a, Supplementary Fig. 2b).

3. The exploration of differences between species is really interesting. Chicken have a body temperature of 40C, would chicken Cathelicidin show more antibacterial activity ? Furthermore, it has been debated that mice have hypothermia instead of fever during infection. Would this go against the hypothesis of the work ? Would mice AMP more active at lower temperature ?

We thank the reviewer for these interesting suggestions. We have now tested the temperature dependence of both murine and chicken cathelicidin. For chicken, its response is indeed shifted: chicken cathelicidin is much more active at 43°C (the fever-temperature for chickens) compared to 40°C (closer to core body temperature) (Supplementary Fig. 3c). In contrast, human cathelicidin's activity peaks at 40°C and does not increase at 43°C (Supplementary Fig. 3b). This further supports our model, since the human febrile temperature is around 40°C.

The case of mice is also interesting, but under some circumstances, mice run a fever when infected (Eskilsson et al., 2021; Murapa et al., 2011), and under others they have hypothermia (Gavin & Satchell, 2017; Granger et al., 2013). So, from this it is not clear what one should expect as far as AMP activity. We found that their cathelicidin (CRAMP) has a maximal activity at their resting/normal body temperature of 37°C, and its activity goes down both at febrile and hypothermic temperatures (Supplementary Fig. 3a). While we do not fully understand this, the difference in temperature-dependence of murine AMPs is consistent with our overall picture, modified by the non-canonical murine response.

4. Page 10, line 154. The authors claim that LL37 and histones have entered the cells. It seems that there are no direct proof of this. The peptides could have as well triggered signalling pathways from the membrane of the macrophages.
Furthermore, is there any indication that LL-37 or histone can binds to mitochondria ? - In addition to this point: How purified/clean are the antimicrobial peptides ? Endotoxin contamination could activate J774.1 macrophages and play a role on mitochondria activation/health.

Both histones and LL-37 have been previously reported to cross cellular membranes (Bankell et al., 2022; Cascone et al., 2012) so they are expected to enter the cells in our experiments. To provide additional support of their entry, we have carried out additional histones and LL-37 imaging and confirm that both enter the cell and co-localize with mitochondria (Supplementary Fig. 4a).

As for AMP purities, histones were isolated from calf thymus and verified through SDS electrophoresis, and LL-37 was synthesized by solid-phase peptide synthesis with reported purity ≥95% by HPLC. Neither source was synthesized by bacteria, obviating a concern for endotoxin contamination.

5. The data on the contact points with LDs is interesting. The microscopy would benefit from a zoomed inset. If the authors' hypothesis are correct, using cold-bloaded animals cell line would not show such a unloading effect. Furthermore, if an active separation LD/mitochondria is happening, what is the mechanism ? Could this be driven by PLINs proteins ? Does heat-shock induce a switch in PLIN expression (PLIN5 to PLIN2 or vice-versa ?) ?
We have now imaged human hepatocyte HepG2 cells because they are flatter and provide higher image quality and lower fluorescence background (Fig. 5a, b). Zoomed insets are also provided. We also imaged human lung epithelial A549 cells and observed a similar trend: a decrease in mitochondria-LD contact at 40°C compared to 37°C (Supplementary Fig. 4c,d), supporting the generality of our findings.

To address the role of PLIN5, we performed immunofluorescence staining for PLIN5 in HepG2 cells under no glucose condition at 37°C and 40°C. We found that the PLIN5-lipid droplet contact ratio slightly decreased at 40°C, but the difference was not statistically significant (Supplementary Fig. 4e,f). Thus, PLIN5 might not be the major reason for the separation of mitochondria and LD at fever temperature.

Finally, we tested cells from Drosophila to address mitochondria-LD contact in a cold-blooded animal, as suggested by the reviewer. In support of our overall picture, we detect no significant change in mitochondria-LD contact between 25°C and 32°C (Fig. 5c, d). Contact at higher temperatures was not assessed due to a significant increase in cell death at these temperatures.

6. Finally, Is there any effect on mitochondrial dynamic (fission/fusion) between 37C and 40C ?
While we did not specifically quantify mitochondrial morphology in this study, we did not observe obvious differences in mitochondrial morphology between 37°C and 40°C in our SIM images (Fig. 5a, magenta channel).

Minor comments:
7. P15: figure 5b is called in the text before Fig 5a
We thank the reviewer for noting this issue. The figure order has been corrected.
8. P9, line 148: I am guessing the macrophages were cultured at 37C and not at room temperature ?
Macrophages were cultured at 37°C not room temperature. The text has been corrected accordingly (line 161).
9. P27, line 477: uM instead of microM
Corrected (line 476).

10. Figure 3 panel E is confusing. Cells have been treated with annexin (575/25 nm excitation and 641/75 nm) but the right graph show Red/Green Fluorescence (575/25 nm excitation and 641/75 nm emission filters for the red channel and 474/27 nm excitation and 525/45 nm emission filters). Are both Red emissions imaged together ? Please explain.
We thank the reviewer for pointing this out. The y-axis should have been 'Annexin V Fluorescence'. We have corrected this error (now Fig. 4e).

11. In general the image magnification is not high enough (figure 3 in particular)
We increased the magnification in Figure 3 (now Figure 4) to improve visualization. We also added zoomed-in insets to Figure 5 to better visualize mitochondria-lipid droplet contacts.

Overall, the manuscript is well written and the basic premise of the study is fundamentally sound. Whilst the level of work can be appreciate, it lacks more robust data to really confirm the findings.

We thank the reviewer for their helpful and detailed suggestions. As they can see, many of them were fruitful and have significantly strengthened the manuscript.

Reviewer #2 (Remarks to the Author):
The manuscript by Yonghan Wu et al. investigates the role of fever-range temperatures (37° C to 40°C) on the efficacy of the human antimicrobial peptide (AMP) LL-37. Using complementary approaches, the authors suggest that hyperthermia enhances LL-37 activity against pathogens without a proportional increase in mitochondrial damage. This provides a compelling evolutionary argument for fever as a host defense mechanism.
While the topic is novel and of high interest to a general audience, several experimental discrepancies and technical omissions must be addressed.

We thank the reviewer for their feedback. We have fully addressed the experimental discrepancies and technical omissions below. In addition, we have performed a number of new experiments and analyses that have increased the rigor and impact of the findings.

Major points

1) Peptide Aggregation and Conformational State: LL-37 is well-known to oligomerize and is highly prone to aggregation, which significantly impacts its biological activity (see Nature Communications 2020, 11:3859 and Biochem J. 2014, 457(3):451-61). Have the authors analyzed how temperature increases specifically alter the aggregation state or secondary structure of LL-37? Can the authors exclude the possibility that the observed "efficiency" is an artifact of experimental buffer conditions favouring a specific oligomeric state that might differ from the complex physiological environment of human macrophages ?

We thank the reviewer for this important suggestion. In response to both reviewers' comments, we looked at the effects of temperature both on LL-37 secondary structure and oligomerization. We found that increasing temperature significantly impacts secondary structure and may increase oligomerization (Fig. 2a and Supplementary Fig. 2b). These findings significantly strengthen the manuscript.

To address the potential dependence of the effect on buffer conditions, we performed an additional assessment in macrophage growth media (DMEM with 10% FBS) and found that consistent with our previous results, temperature increases membrane permeabilizing activity (Supplementary Fig. 1a).

Further, we note that while bacteria were treated in buffer, the mitochondrial damage from LL-37 and histones occurred inside an intracellular environment (Fig. 4a-d), further addressing the reviewer's concern of potential buffer effects.

2) Inconsistencies in Imaging and Quantitation (PI Assay): The representative images for membrane permeabilization (Propidium Iodide entry) are not convincing and appear to contradict the quantified data:
Figure 1b vs. 1d: In Fig 1b (E. coli + LL-37 at 37°C), the fluorescence is invisible, yet the reported PI/Area is around 8,000-10,000. In contrast, Fig 1d (LL-37 + Histone) shows clearly visible fluorescence, yet the mean PI/Area is reported as only 5,000.
Figure 2a vs. 2b: For Pelteobagrin, a PI/Area of 6,000- 7,000 is reported for both panels, yet the signal is clearly visible in 2a and virtually absent in 2b (35°C).
The authors must provide truly representative images or explain these numerical discrepancies.
We thank the reviewer for pointing out this issue. We had not taken into account that there might be intensity display differences between different panels in the same figure and agree that the images were confusing. We have now updated the images to use comparable lookup tables across different panels within the same figure. The quantifications were accurate and are unchanged.

3. Discrepancies between Text and Data (Cold-blooded AMPs): There are some contradictions in the reporting of the antimicrobial activity (CFU counts) in Figure 2:
Pelteobagrin: The text and also fluorescence PI assay states activity decreases with temperature, but Figure 2e appears to show an significant increase in activity (CFU counts) as temperature rises.
Magainin: The data does not support a temperature-dependent decrease in activity. CFU counts only appear to change at 42°C, with no significant trend at intermediate (37°C) temperatures.
The authors need to carefully re-examine their figures and ensure the text accurately reflects the shown results.

We thank the reviewer for raising this issue. We have now clarified in the text that PI and CFU assays measure different effects, specifically that PI measures membrane permeability and CFU measures bactericidal activity, and added additional statistical analyses. Increased membrane permeability is expected to roughly correlate inversely

with CFUs but this activity alone is not the sole factor responsible for bacterial death. From our previous work (Duong et al., 2025), we have found that other contributing factors include induced membrane pore size, stability of pores, and effects on intracellular mechanisms that affect bacterial viability. We have assessed our findings in this context.

For magainin, increasing temperature steadily decreased both membrane permeability (PI) and bactericidal activity (CFU) (Fig. 3a, b). An additional analysis shows that a statistically significant change in CFU can be observed as low as 32°C. PI and CFU have roughly an inverse relationship for OH-CATH as well (Fig. 3c, d). For pelteobagrin, we agree that the inverse PI and CFU trend is not followed (Fig. 3e, f). This is interesting and suggests that the membrane permeabilizing activity of pelteobagrin is not sufficient for bactericidal activity. Our previous studies (Doolin et al., 2020; Duong et al., 2025) showed that multiple mechanisms from AMPs contribute to bactericidal activity, including targeting intracellular machinery and inhibiting transcription. It is possible that pelteobagrin has multiple mechanisms whose activity have different temperature dependences. We have added a discussion of this in the revised manuscript (lines 326-335).

4) Synergy Limitations: In the synergistic assays, only a single concentration combination was tested for the two molecules. The authors must justify how these specific concentrations were chosen and demonstrate their physiological relevance within a cellular environment.

In synergistic assays, we used histone concentrations in the range of 5-10 μg $ml^{-1}$and LL-37 in the range of 5-40 μg ml^{-1} (i.e., Fig. 1d, e and Supplementary Fig. 1c, d). These concentrations were based on a series of LL-37/histone combinations performed in (Duong et al., 2025) (Figure S7a-c). In particular, 10 μg ml^{-1} histones + 20 μg ml^{-1} LL-37 resulted in a significant decrease in CFU and 12 h lag for growth curve. In regards to physiological relevance, these concentrations are comparable to the range measured *in vivo*. In particular, LL-37 is ~5 μg ml^{-1} in healthy infants (Barlow et al., 2006) and can be upregulated to 30 μg ml^{-1} during infections (Keutmann et al., 2022). Histones can reach a concentration of 3 μg ml^{-1} in sepsis patients (Wildhagen et al., 2015) and exceed 50 μg ml^{-1} in ARDS patients (García-Gimenez et al., 2025). We have added these justifications to the Results in lines 62-66.

5) Quantification of Mitochondrial Damage (Lines 160–166). The methodology used to calculate the fold increase in mitochondrial damage is not clearly described. The values provided (1.2-fold and 1.7-fold) appear to derive from two distinct experiments measuring different biological phenomena using different fluorescent probes. Absolute fold-change values can only be directly compared if a precise correlation exists between the underlying physiological phenomenon and the specific sensitivity/linear range of each probe.

We thank the reviewer for this comment. We have clarified our approach in both the Method and Main Text sections. Specifically, mitochondrial damage was quantified using fold changes in the JC-1 red/green fluorescence ratio relative to the indicated baseline condition. We acknowledge that mitochondrial damage and bacterial membrane permeabilization were measured using two different probes. We revised the text accordingly to avoid direct quantitative comparison.

6) Quantification of Annexin V (Figure 3): The authors must clarify the biological and physical significance of the 'Red/Green Ratio' reported in Figure 3. Annexin V and PI are independent markers for distinct cellular events (membrane inversion vs. nuclear membrane rupture). Therefore, a ratio between these two signals is unconventional and lacks a standardized scale.

We thank the reviewer for finding this error. The y-axis should have been ‘Annexin V Fluorescence’. We have corrected this error (now Fig. 4e).

Minor Points

7) Line 125: Define the specific baseline/control groups used to calculate the "fold increase" in PI-positive cells in Figure 2d.

We now describe the baseline groups that were used to calculate fold-increase for each peptide in figure captions (now Figure 3g).

8) Line 94: Note that OH-CATH is a cathelicidin like LL-37. Discussing their sequence differences could explain their divergent thermal performance.

We thank the reviewer for this suggestion. We performed additional experiments for the revision, finding that chicken CATH-1 and mice CRAMP also exhibited temperature-dependent antimicrobial activity. We thus performed a sequence comparison between four cathelicidins: chicken CATH-1, mice CRAMP, LL-37 and OH-CATH (Supplementary Fig. 3d,e). We have identified conserved residues and key residues that diverge from LL-37. We suggest that these residues could be involved in determining temperature sensitivity in cathelicidins (lines 270-280).

9) Figure 1: State the incubation times for all antimicrobial assays; this is essential for interpreting membrane permeabilization kinetics.

We have now clarified incubation times for all antimicrobial assays in both the figure captions (line 723) and Methods section (lines 471-474).

10) Line 129: Please, standardize all peptide concentrations. Currently, Magainin is in µM while others are in µg/ml.

We have now standardized all peptide concentrations according to mass/volume.

Author responses in blue

Reviewer #1 (Remarks to the Author)

The authors have answered all my comments and have made their conclusion cleary more robust. This is a very nice piece of work that will hopefully lead to more interesting discoveries.
Congratulation on the authors for this nice work and their great effort to answer all queries from reviewers.(Remarks on code availability)

I do not have the expertise to review the code

We thank the reviewers for their positive assessment.

Reviewer #2 (Remarks to the Author):
In this revised manuscript, the authors explore how elevated temperature enhances the activity of human LL-37 and histones, proposing a physiological role for fever in mammalian host defense.

The authors have addressed several initial concerns by performing additional experiments, which are now included in the Supplementary Information. While these new additions clarify certain aspects, several important technical and presentation issues remain:

1.Figure Organization and Logical Flow:
Heavy reliance on Supplementary Figures—discussed with equal weight to main figures, forces the reader to constantly flip between documents. Reorganizing the main and supplementary figures is essential to consolidate related data within single panels, ensuring the authors' rationale and scientific narrative remain coherent without page-switching.
We have moved a number of Supplementary figures to the main figures to reduce this issue. We moved Supplementary Fig. 1b to main Fig. 1f, Supplementary Fig. 2c and d to main Fig. 2d, e. We reorganized other Supplementary Figures to maintain a coherent flow.

2.Biophysical Interpretation and Structural Data (Figures 2 & S2):

a) LL-37 CD Spectral Interpretation (Figure 2): The authors attribute increased activity at higher temperatures to a structural transition based on Far-UV CD spectra. However, the characteristic α-helical minima (208 nm and 222 nm do not shift position, and signal variation is minimal. Minor ellipticity changes without peak shifts typically reflect temperature-dependent solvent dynamics rather than secondary structure alterations.

We thank the reviewer for the comment. We agree that LL-37 shows characteristic α-helical bands at both 37°C and 40°C, suggesting that there is no transition in

predominant secondary structure type. However, the α-helical content does change with temperature. The amplitude of the three characteristic bands all shifted with temperatures: the 208 nm from -16.73 to -16.15 mdeg, 222 nm from -12.24 to -10.63 mdeg, and the peak at 193 nm decreased from 15.36 to 10.6 mdeg. We further estimated the helix content using BeStSel (Micsonai et al., 2018). The α-helical content decreased from 45.83% at 25°C to 34.04% at 37°C and 29.53% at 40°C. Moreover, the antiparallel β-sheet content rose from 3.08% (25°C) to 9.04% (37°C) and to 11.38% (40°C). The shift in the amplitude of these bands has been used to estimate helical content changes in other studies (Al-Adwani et al., 2020; Chen et al., 2002). While this data does not exclude a role for temperature-dependent solvent dynamics, these data are consistent with the interpretation that temperature alters helical content. We have revised the text accordingly (line 88-92).

b) Histone H1 CD Spectrum (Figure S2e): The reported CD spectrum for histone H1 appears characteristic of a fully alpha-helical protein. In free solution, full-length H1 is dominated by its disordered tails and exhibits a high random-coil content !. The authors should clarify the buffer conditions or construct used.

Unfractionated histone, which contains a mixture of core and linker histones, were used for the CD spectrum (this is now Fig. S2c). We have made the histone forms and buffer conditions clearer in the Methods, Main text, and Figure captions.

c) C) Inconsistent Oligomerization and "Memory Effect" (Figure S2b): No oligomerization differences are observed between 37° C and 40° C (supplementary Fig. S2b), which contradicts the claim that LL-37 retains a 45-minute "memory effect" after incubation at 40 ° C. Because conformational relaxation of a 37-residue peptide occurs on microsecond timescales, a 45-minute kinetic lag requires a plausible physical rationale (e.g., slow assembly/disassembly kinetics or metastable aggregation).

We thank the reviewer for the comment. While the differences between oligomerization at 40°C vs 37°C is modest, it is statistically significant. We have now repeated the cross-linking experiment in additional biological triplicate and consistently observe higher signal from LL-37 oligomers at 40°C compared to at 37°C (Supplementary Fig. 2b, with images placed in Source Data file and repository under Data Availability). Specifically, Kolmogorov-Smirnov tests on signal intensity of three replicates in Supplementary Fig. 2b yielded a p value of 0.026. This indicates a small but statistically significant increase in oligomerization signal across replicates. We also noted that the memory effect may not be purely attributed to oligomerization of LL-37. Membrane lipids can stabilize the LL-37 oligomers (Sancho-Vaello et al., 2020) and metastable aggregation may not be captured by the crosslinking method but both could contribute to the memory effect. We have modified the oligomerization discussion to reflect this (line 104-109).
